# Comparative Transcriptomics of Fusarium graminearum and Magnaporthe oryzae Spore Germination Leading up To Infection

**DOI:** 10.1128/mbio.02442-22

**Published:** 2023-01-04

**Authors:** Cristina Miguel-Rojas, Brad Cavinder, Jeffrey P. Townsend, Frances Trail

**Affiliations:** a Department of Plant Biology, Michigan State University, East Lansing, Michigan, USA; b Department of Biostatistics, Yale School of Public Health, New Haven, Connecticut, USA; c Department of Ecology and Evolutionary Biology, Yale University, New Haven, Connecticut, USA; d Program in Computational Biology and Bioinformatics, Yale University, New Haven, Connecticut, USA; e Program in Microbiology, Yale University, New Haven, Connecticut, USA; f Department of Plant, Soil and Microbial Sciences, Michigan State University, East Lansing, Michigan, USA; Universidad de Córdoba

**Keywords:** *Fusarium graminearum*, *Magnaporthe oryzae*, barley infection, infection process, secondary metabolites, spore germination, transcriptome

## Abstract

For fungal plant pathogens, the germinating spore provides the first interaction with the host. Spore germlings move across the plant surface and use diverse penetration strategies for ingress into plant surfaces. Penetration strategies include pressurized melanized appressoria, which facilitate physically punching through the plant cuticle, and nonmelanized appressoria, which penetrate with the help of enzymes or cuticular damage to breach the plant surface. Two well-studied plant pathogens, Fusarium graminearum and Magnaporthe oryzae, are typical of these two modes of penetration. We applied comparative transcriptomics to Fusarium graminearum and Magnaporthe oryzae to characterize the genetic programming of the early host-pathogen interface. Four sequential stages of development following spore localization on the plant surface, from spore swelling to appressorium formation, were sampled for each species on culture medium and on barley sheaths, and transcriptomic analyses were performed. Gene expression in the prepenetration stages in both species and under both conditions was similar. In contrast, gene expression in the final stage was strongly influenced by the environment. Appressorium formation involved the greatest number of differentially expressed genes. Laser-dissection microscopy was used to perform detailed transcriptomics of initial infection points by F. graminearum. These analyses revealed new and important aspects of early fungal ingress in this species. Expression of the trichothecene genes involved in biosynthesis of deoxynivalenol by F. graminearum implies that toxisomes are not fully functional until after penetration and indicates that deoxynivalenol is not essential for penetration under our conditions. The use of comparative gene expression of divergent fungi promises to advance highly effective targets for antifungal strategies.

## INTRODUCTION

Fusarium graminearum and Magnaporthe oryzae are two of the most important pathogens of staple food crops worldwide (1, 2). Their respective destruction causes 3.3% annual loss in wheat harvests and 4.3% annual loss in rice harvests around the world (3–5). F. graminearum is the causal agent of Fusarium head blight (FHB) of wheat and barley and stalk rot of corn. An important component of the pathogenicity of F. graminearum is its secretion of the mycotoxin deoxynivalenol (DON) during fungal colonization (6, 7). DON functions as a pathogenicity factor and accumulates in infected grain (8), rendering the grain unfit for human and animal consumption (9). Development of even moderately resistant cultivars has proven difficult, and the high tolerance of F. graminearum to extant fungicides has made exogenous control difficult (10, 11).

M. oryzae causes rice blast, a devastating disease throughout Asia. There are highly effective resistance genes known in rice to combat M. oryzae. However, current resistance genes are not durable or broad spectrum, traits that are essential to successful long-term control (12). These fungi manifest disease with quite different approaches, but they share resilience to changing climatic conditions (13). Furthermore, the probability of disease-causing strains migrating due to global climate change (14) highlights the importance of better elucidating the very early steps of disease initiation.

Historically, Fusarium and *Magnaporthe* species have been models for understanding the mechanisms of ingress into plants. Spores are responsible for initiation and propagation of the majority of biotic plant diseases, including diseases caused by M. oryzae and F. graminearum (15–19). Spore germination is a promising target for antifungal strategies. Once the fungus has infiltrated host tissue, the pathogen is much harder to eliminate. Spore germination is the first step toward establishing many fungal diseases (20). Nevertheless, the genetics of spore germination have not been systematically or comparatively analyzed across multiple fungal species. The formation of simple appressoria completing direct penetration by using enzymes was first elucidated in F. solani (21–23). Recent studies have highlighted the contrasting approach used by melanized appressoria in M. oryzae, in which pressure within the appressoria is built up and drives penetration (24–28). However, to initiate disease, fungal spores must arrive on a compatible plant surface, germinate, differentiate an infection structure at the proper location, and then achieve ingress and colonization (29–33). Hyphal growth from germination to appressorium formation is more extensive in F. graminearum than in M. oryzae, resulting in greater presence of fungal tissue on the plant surface in the former. F. graminearum germlings locate and interact with trichomes before differentiating hyphopodia consisting of several appressoria (6, 16, 34). In contrast, M. oryzae germinates to form a short germ tube then generates a simple appressorium (35, 36).

Previous studies of each of these two pathogens have analyzed transcriptomics across infection time intervals. Investigations of F. graminearum have combined gene expression analysis with microscopic observation, typically focusing on the progress of hyphal growth postinfection. Brown et al. (37) investigated symptomless infections from samples collected 7 days postinfection (dpi). Mentges et al. (7) documented gene expression in isolated infection cushions and runner hyphae, collecting infection time points beginning at 6 h postinfection through 3 days, and identified the upregulated genes characteristic of each of these structures without distinguishing the results of each time point. Several investigations of the interactions of F. graminearum with susceptible and resistant wheat lines were sampled at 3 dpi (38–40). In addition, Bonnighausen et al. (41) performed metabolic profiling of the infected wheat rachis to document a role for DON in susceptibility. Expression during conidial germination in F. graminearum was investigated on agar using Affymetrix GeneChip technology (30), which, as a first look at initial germination of these spores on medium, identified many categories of metabolic genes and gave a detailed account of genes involved in peroxisome maturation.

Transcriptomic analyses of conidial germination and appressorium development by M. oryzae have included studies on artificial surfaces that induce appressorium development (42, 43) and on rice, which include disease development (44, 45). Several studies (44–47) focused on gene knockouts to study development after appressorium formation was initiated. Mosquera et al. (44) isolated infection hyphae in host cells and identified effectors, which are generally expressed during plant ingress, whereas Shimizu et al. (45) focused on broad metabolic gene expression and expression of *avr* genes, including knockouts.

Here, we present transcriptomic analyses from spore arrival on the plant surface to germination and the formation of appressoria in these two species. To provide comparisons for responses to distinct growth conditions, we cultured these species on a defined medium and through host infection. To extend our study into the initial stages of plant penetration of F. graminearum, we used laser-dissection microscopy to isolate and then perform transcriptomics at the first stages of host penetration. Close comparative characterization of the very early infection processes of these two pathogens on a common host and a common medium enabled us to tease apart some of the mechanisms that differentiate the two infection strategies. In particular, our comparative analysis revealed evidence of the adaptations of gene expression that were concomitant with the evolution of one type of penetration (use of pressurized appressoria) over the other (use of simple appressoria), a key component of broad-based comparative study of the transcriptomic and phenotypic evolution of fungal diversity in sexual and asexual sporulation (48–54).

## RESULTS

### Four stages of conidial germination on Bird medium and on barley sheaths.

**FIG 1:**
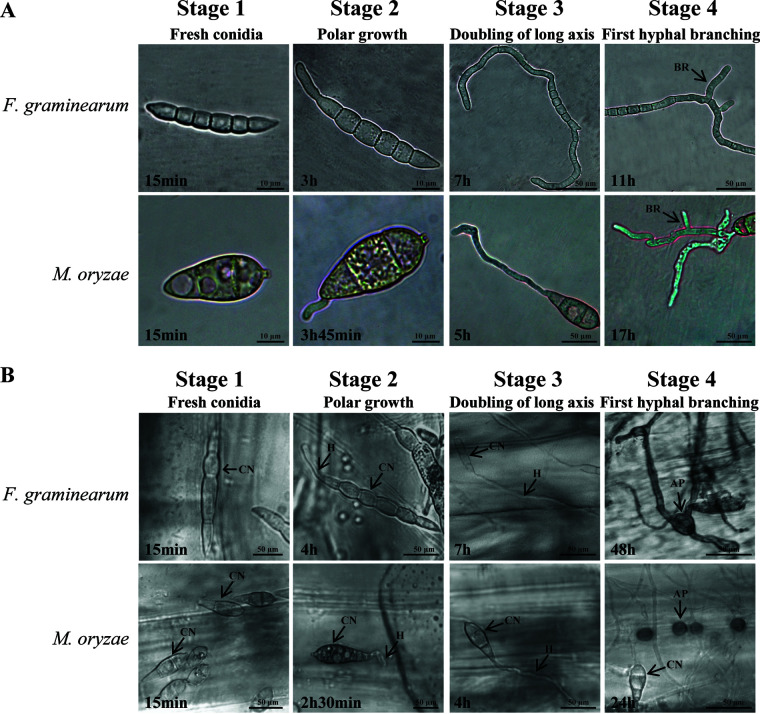
(A and B) Stages of conidial germination and development for transcriptome analysis on (A) medium (A) and host (barley leaf sheaths) (B). Time indicates the point at which peak numbers of conidia reach the indicated stage; AP, appressorium; CN, conidium; BR, hyphal branch; H, hypha; stage 1, fresh conidia; stage 2, polar growth; stage 3, doubling of long axis; stage 4, first hyphal branching (Bird medium) or appressorium formation (host).

Spore germination was characterized both on Bird medium and on host plants (barley leaf sheaths), and samples were collected to complete mRNA sequencing for each stage and species. Using light microscopy, stages 1 to 4 of conidial germination were delineated in F. graminearum and M. oryzae (Fig. 1): stage 1, fresh conidia directly obtained from actively growing cultures; stage 2, initiation of polar growth of the germ tube; stage 3, doubling of the long axis of the germ tube, and stage 4, initiation of the first hyphal branch on Bird medium or the formation of the appressorium on the host. Approximately 80% of wild-type F. graminearum and M. oryzae conidia produced their first polar growth (stage 2) within 3 h following inoculation onto Bird medium (Fig. 1A). The majority of F. graminearum germlings reached stage 3 on medium by 7 h, whereas the majority of M. oryzae germlings reached this developmental stage by 5 h. M. oryzae can be induced to produce appressoria on artificial hydrophobic surfaces (55). However, Bird medium is not appressorium-inductive for *M oryzae.* In contrast to germination on medium, conidial germination and development on host plants progressed faster in M. oryzae than in F. graminearum—particularly during appressorium formation, where F. graminearum required 24 h more than M. oryzae to reach peak percentage of germinated conidia with penetration structures (Fig. 1B).

### mRNA sequencing during conidial germination and plant penetration.

A total of 20 to 114 million 50-bp single-end reads were obtained from each developmental stage collected for F. graminearum and 21 to 82 million 50-bp single-end reads from each M. oryzae stage. The F. graminearum data set on Bird medium was analyzed previously for long noncoding RNA content and is available through Gene Expression Omnibus (GEO) series accession number GSE109088 (56). After processing and removal of poor-quality and short-length raw reads, 93.0% (M. oryzae) and 98.0% (F. graminearum) of reads from Bird medium samples mapped to the fungal reference genomes (Table S1 in the supplemental material). For both fungal species, reads from *in planta* samples that mapped to the fungal reference genomes increased from stage 2 to stage 4 (Table S1), with mapped reads at stage 1 having the highest percentage at 59%. In contrast, numbers of mapped fungal reads remained roughly consistent along the time course in the Bird medium samples (Table S1).

10.1128/mbio.02442-22.4 TABLE S1 Summary of transcriptome sequencing results for F. graminearum and M. oryzae on medium and on the host. Download Table S1, XLSX file, 0.04 MB. Copyright © 2023 Miguel-Rojas et al. 2023 Miguel-Rojas et al. https://creativecommons.org/licenses/by/4.0/ This content is distributed under the terms of the Creative Commons Attribution 4.0 International license.

**FIG 2:**
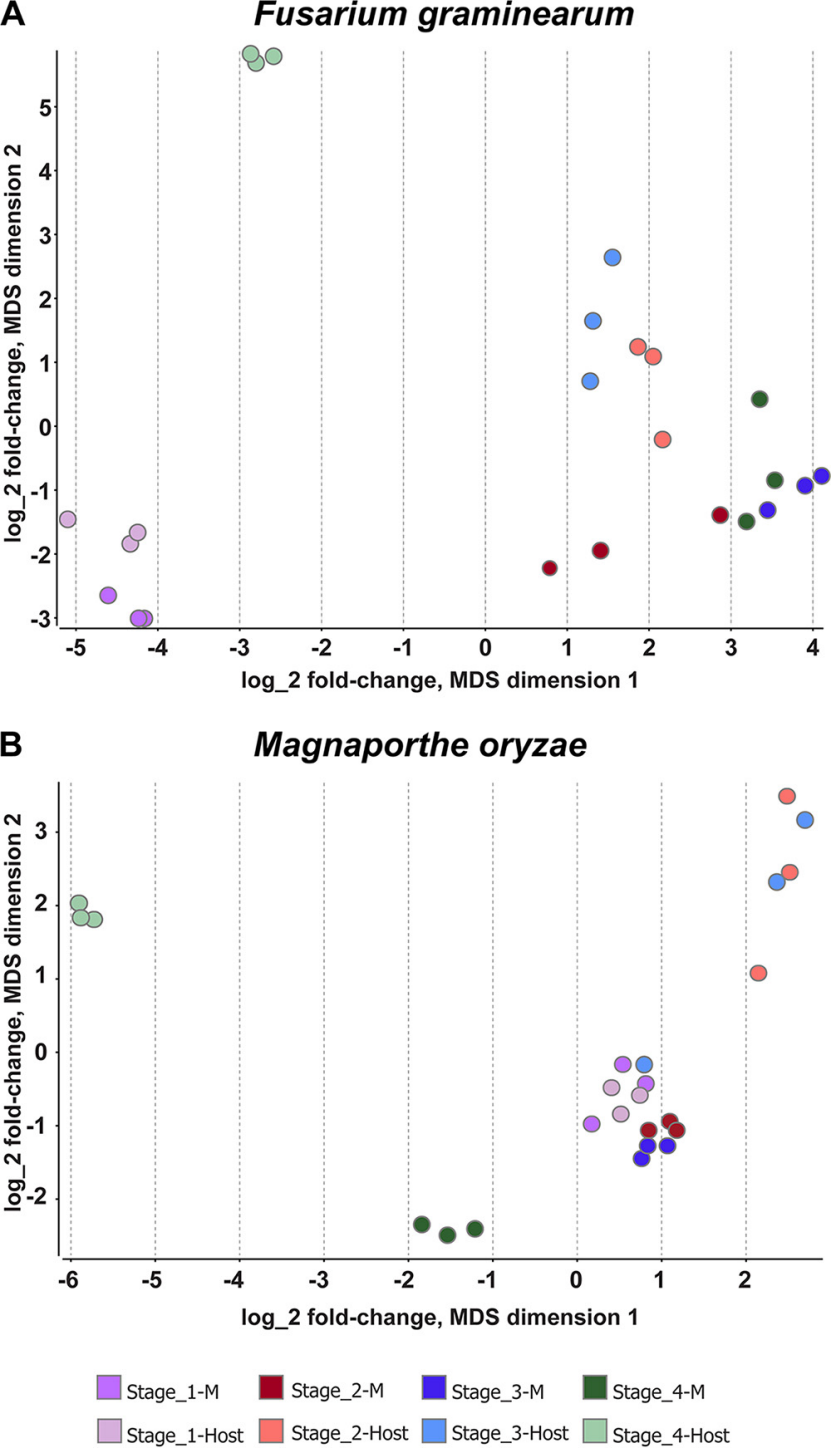
Multidimensional scaling (MDS) analysis plot applied to the transcriptomes of conidial germination on medium and on the host. (A) F. graminearum. (B) M. oryzae. Each point represents one sample, and the distance between two points reflects the leading log_2_ fold change of the corresponding RNA-seq samples. Plot dimensions 1 and 2 show samples from medium and from the host at different stages, resulting in separate clusters.

Multidimensional scaling (MDS) analysis allows the visual comparison of the levels of similarity of different parts of a data set (57). We used MDS to visualize and compare gene expression between biological replicates of stages 1 through 4 on the host and on Bird medium in both species (Fig. 2). We also sought to identify possible outliers. Clustering of samples in both species and under both growth conditions showed that biological replicates are grouped in close proximity, validating the integrity of our RNA sequencing. Moreover, the analysis revealed the relative similarity of stages 1 to 3 when compared between the host and Bird medium samples. In contrast, the same comparison for stage 4 exhibited substantial divergence. Stage 4 on medium was similar to the other stages on medium, whereas stage 4 on the host exhibited pronounced transcriptional differences from the other host samples.

### Transcriptional reprogramming during spore germination on the host.

**FIG 3:**
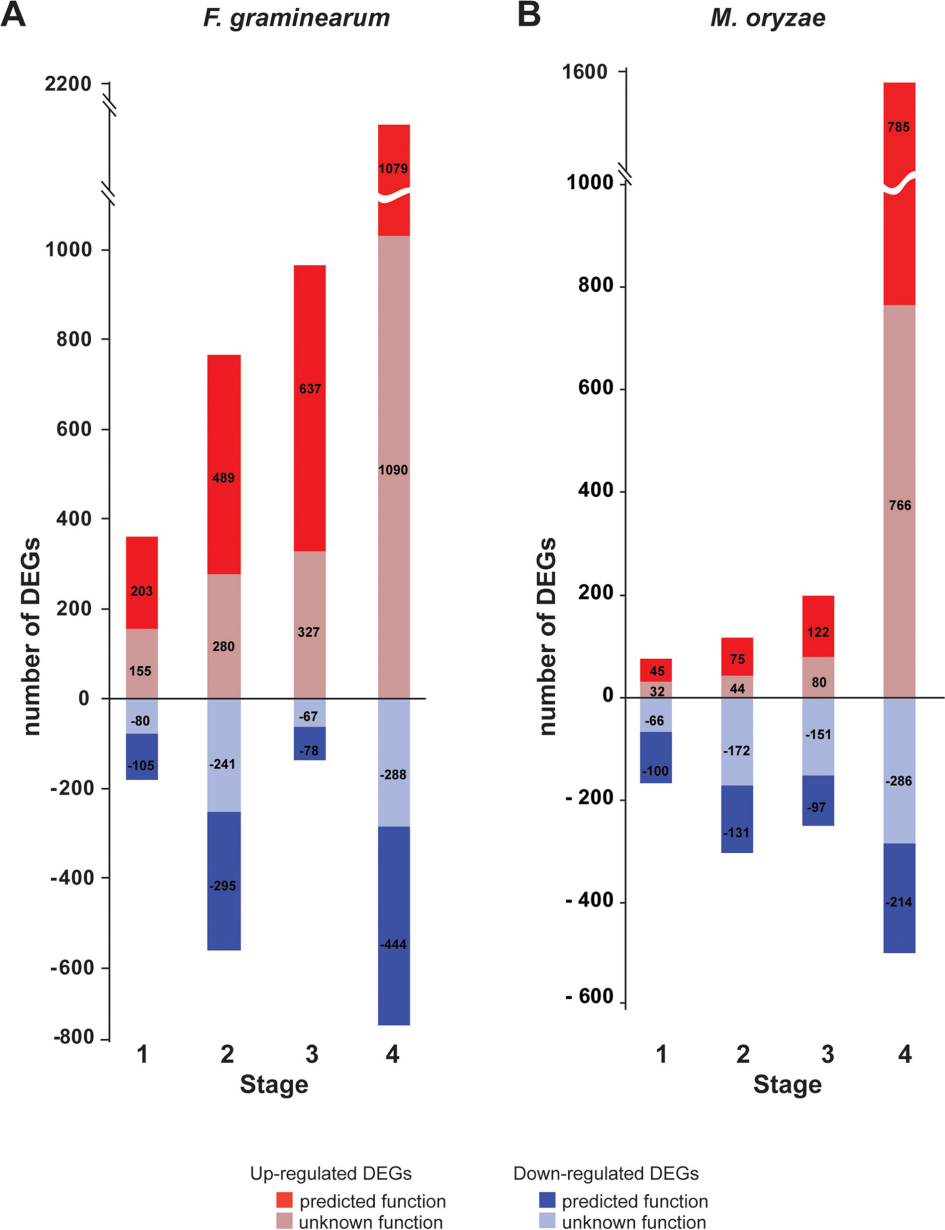
Distribution of differentially expressed genes (DEGs; >3-fold; 5% false-discovery rate) with predicted and unknown functions in each stage on the host. (A) F. graminearum. (B) M. oryzae.

Analysis of gene expression across stages 1 to 4 was performed to compare development on a defined medium versus on host tissue and to better identify affected genes. Distributions of genes that increased or decreased in expression (>3-fold; 5% false-discovery rate [FDR]) in each stage on the host were compared between the two species (Fig. 3). In F. graminearum, numbers of upregulated genes increased progressively from stages 1 to 4, with stage 4 containing the largest number of upregulated genes of unknown function. In M. oryzae, a different trend was observed, showing fewer genes expressed in stages 1 to 3 than in stage 4 and altogether fewer genes expressed at each time point than in F. graminearum. This observation in M. oryzae may be consistent with previous studies that demonstrated that during spore germination on inductive surfaces (such as the hydrophobic host), the spores undergo autophagy as germination progresses, and gene expression is reduced (58, 59). Both species have fewer numbers of downregulated genes than upregulated genes across all intervals, perhaps consistent with increasingly complex development of new structures.

For genes with stage-specific expression patterns, significant functional enrichment (*P < *0.05) was identified for FunCat pathways across the morphological stages in F. graminearum and M. oryzae on the host (Table S2A and B). In particular, the initial two stages (fresh conidia and polar growth) of F. graminearum showed significant enrichment (*P < *0.05) of anabolic processes (carbon, nitrogen, and fatty acid metabolism, biosynthesis of amino acids, energetics, cellular transport, transport facilitation and transport routes, and biogenesis of cellular components), involving a large proportion of genes (1,093). These categories reflect the biological transition from conidia to maintenance of polar growth, where hyphal tips undergo continuous elongation. Interestingly, expression of genes associated with cell rescue, defense, and virulence were enriched for the first time at stage 2, suggesting response to the host. Stage 3 (doubling of long axis) requires more cellular components and fungal tissue production because hyphal elongation continues. In line with those biological needs, stage 3 presented 874 significantly enriched genes (*P < *0.05), with a notable number of them associated with cellular transport, transport facilitation, and transport routes compared with stage 2. In addition, for the first time, expression of genes associated with interactions with the environment and secondary metabolism were significantly enriched at this stage. In F. graminearum, deoxynivalenol genes began to be expressed during this stage. Finally, at stage 4, one new significantly enriched category appeared, cell fate, anticipating infection cushion formation and appressorium formation.

**FIG 4:**
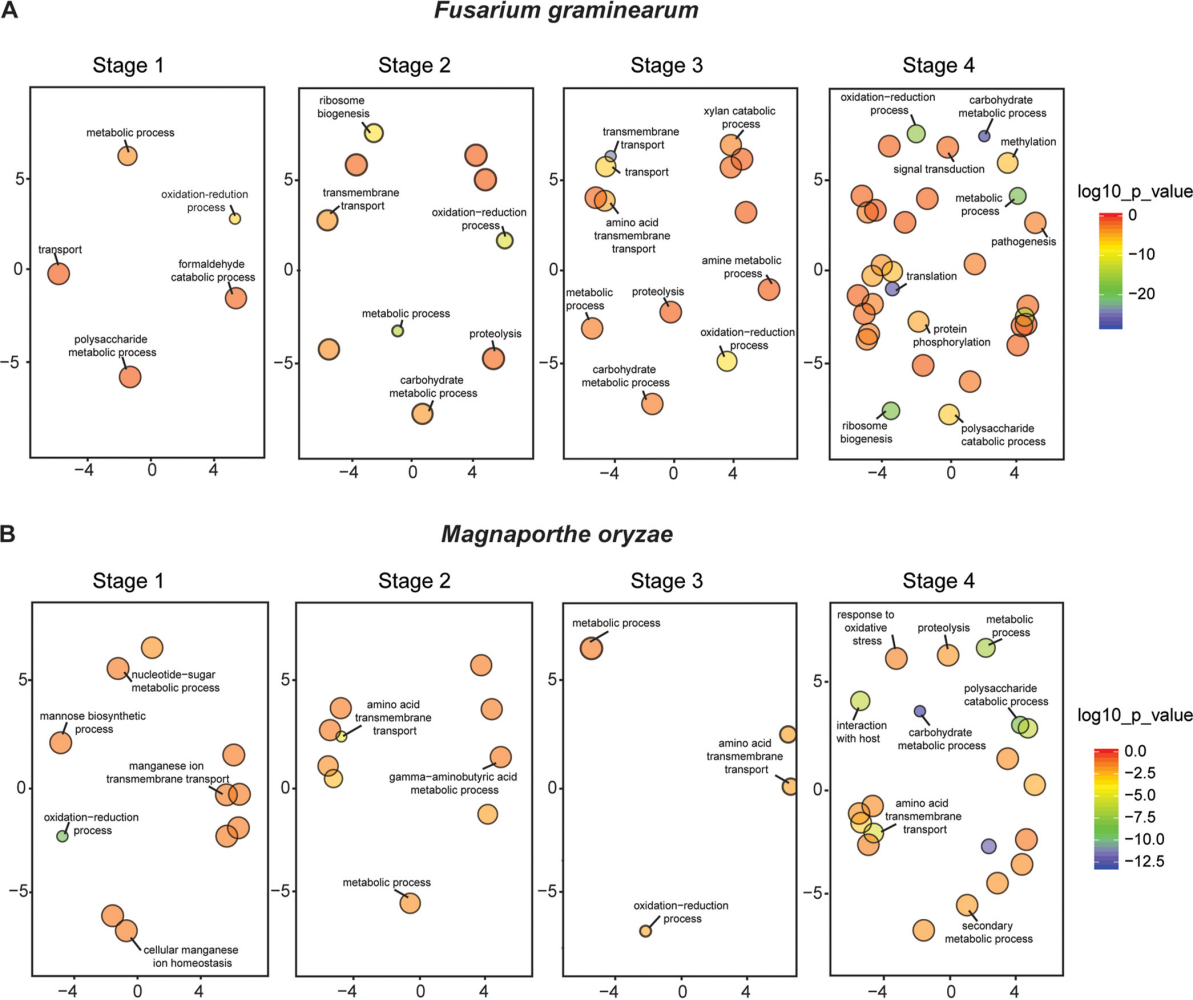
Comparison of functional enrichment of DEGs in the host for F. graminearum (A) and M. oryzae (B). DEGs (>3-fold for F. graminearum and > 2-fold for M. oryzae) were assessed for degree of functional enrichment and plotted in two-dimensional semantic spaces. Only GO terms with a *P *value of <0.05 are depicted in each panel. The size of the circles indicates frequency of GO term in the underlying Gene Ontology annotation database (circles of more general terms are larger). The color scale indicates the log_10_ of the *P* value. See Table S3 for fully detailed data of GO terms.

10.1128/mbio.02442-22.5 TABLE S2 (A) Functional enrichment analysis for genes exhibiting significant host-specific expression patterns in F. graminearum; adjusted *P* value of <0.05. (B) Functional enrichment analysis for genes exhibiting significant host-specific expression patterns in M. oryzae; adjusted *P* value of <0.05. (C) GO terms enriched in F. graminearum and plotted in REVIGO (Fig. 4). (D) GO terms enriched in M. oryzae and plotted in REVIGO (Fig. 4). Download Table S2, XLSX file, 0.3 MB. Copyright © 2023 Miguel-Rojas et al. 2023 Miguel-Rojas et al. https://creativecommons.org/licenses/by/4.0/ This content is distributed under the terms of the Creative Commons Attribution 4.0 International license.

In M. oryzae, the number of significantly upregulated genes (304; *P < *0.05) was quite similar to the number of significantly downregulated genes (399) in stages 1 to 3, indicating a limited diversity of processes necessary for appressorium formation. Stage 4 exhibited significantly enriched categories similar to the ones reported in F. graminearum, such as metabolism, energy, cellular transport, transport facilitation and transport routes, and cell rescue, defense and virulence. The appressorium of M. oryzae together with the initiation of the biotrophic phase of infection likely explain this enrichment.

Gene Ontology (GO) enrichment analysis was performed for the stage-specific differentially expressed genes (DEGs), reflecting different key biological processes associated with germination on the host (Fig. 4; Table S2A and B). In F. graminearum, the genes involved in each stage increased in the number of categories as development progressed. Stages 1 to 2 were enriched in GO pathways related to anabolic processes, including transmembrane transport, ribosome biogenesis, and metabolic processes, which are involved in the growth of the fungus. Stage 3 was the first stage to have genes significantly enriched for GO terms related to polysaccharide catabolism (enzymes for degradation of the host wall), indicating that the recognition and interaction with the host was already established. In stage 4, genes were significantly enriched for GO terms related to pathogenesis and posttranslational modification of proteins, including methylation and phosphorylation, which are key for signal transduction during the infection process (60–62). In M. oryzae, enriched GO terms at stages 1 to 3 were mainly related to transport, which could indicate a rapid mobilization of components required for appressorium development. Stage 4 was enriched in GO terms related to interaction with the host, polysaccharide catabolism, and secondary metabolism, among others, which are directly related to the infection process.

10.1128/mbio.02442-22.6 TABLE S3 (A) Upregulated genes identified in stage 5 in F. graminearum. (B) The top 100 upregulated genes in M. oryzae expressed on host at stage 4. Download Table S3, XLSX file, 0.2 MB. Copyright © 2023 Miguel-Rojas et al. 2023 Miguel-Rojas et al. https://creativecommons.org/licenses/by/4.0/ This content is distributed under the terms of the Creative Commons Attribution 4.0 International license.

### Transcriptomics of laser-captured trichomes and epidermal cells.

**FIG 5:**
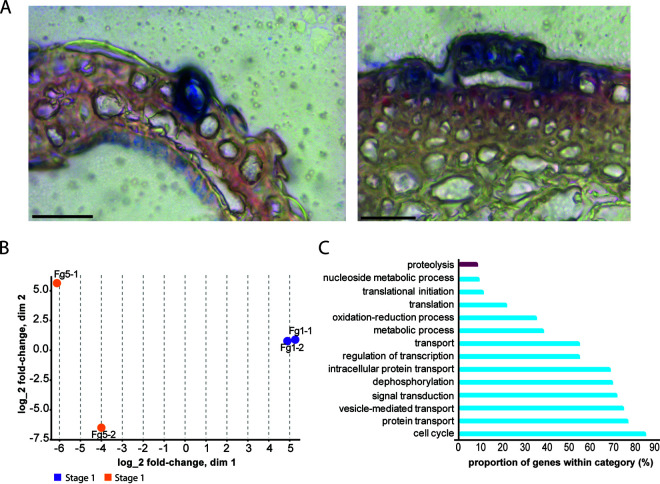
Laser dissection of stage 5 F. graminearum infection sites on barley paleae and transcriptomics analysis. (A) Representative laser-captured infection sites stained with toluidine blue (hyphae) and Safranin O (host cell walls). Left, hyphal penetration and colonization of a single trichome. Right, infection and colonization of epidermal cells (surface) and reaction of sclerenchyma cells (below surface) to ingress of fungus. Lignified cell walls stained red with Safranin O; scale bar, 30 μm. (B) Multidimensional scaling (MDS) plot showing the expression correlation of the stage 1 and stage 5 samples; dim, dimension. (C) Gene Ontology (GO) enrichment analysis of biological processes for upregulated genes (purple) and downregulated genes (blue) of samples in B.

Transcriptomics was applied to laser-capture microscopy to better understand the interactions of F. graminearum with the surface cells of barley lemmas and paleae during infection. Infection sites were stained with toluidine blue (hyphae) and Safranin O (host cell walls), which aided in their identification among the surface cells. Cells at the appropriate infection stage were removed as individuals or in groups by laser capture. About 30 to 40% of these cells included infected trichomes; the rest were colonized epidermal and mesophyll cells within which coiled intracellular hyphae were clearly visible in the lumen (Fig. 5A). Areas where several cells were infected exhibited staining suggesting the presence of lignification in the host cells (Fig. 5A, right). Two pooled samples, consisting of 516 and 525 captured regions, were generated, and mRNA was isolated and sequenced. A total of 8.5 million 50-bp single-end reads were obtained from each sample. The analyses of these sequences are presented in Fig. 5B and C.

MDS analysis was performed for stage 1 growth on the host and was compared with laser dissections of the initial infections of host cells, which we call stage 5 (Fig. 5B). A volcano plot was generated to illustrate the distribution of genes up and downregulated in stage 1 versus stage 5 and comparison of stages 2 to 4 on the host versus stage 5 (Fig. S1). The most notable finding showed that the GO category proteolysis is significantly upregulated between stage 1 and stage 5 (Fig. 5C). All proteins encoded by upregulated genes were assessed for signal peptides using SignalP (http://www.cbs.dtu.dk/services/SignalP) and for nonclassical secretion using SecretomeP (mammalian; http://www.cbs.dtu.dk/services/SecretomeP). Thirty-one of 89 proteins had an N-terminal signal peptide sequence (Table S3A) predicted by SignalIP (63), indicating that they are likely secreted proteins. Moreover, SecretomeP (64) predictions for 18 proteins suggested that these might be secreted in a nonclassical way (Table S3A). Overall, 55% of upregulated genes showed secretion signal peptides, a pool of the secretome that could play important roles in pathogenicity. Furthermore, some of these genes encode carbohydrate-active enzymes (CAZymes), many of which are likely involved in plant cell wall degradation, and possible virulence factors (Table S3A).

10.1128/mbio.02442-22.1 FIG S1 Differentially expressed genes in successive developmental stages compared to laser-dissected cells at stage 5 are revealed in volcano plots. Upregulated genes in the advanced stages are highlighted red, while downregulated genes are highlighted green. Black indicates genes not significantly differentially expressed; false-discovery rate < 0.05. Download FIG S1, TIF file, 2.5 MB. Copyright © 2023 Miguel-Rojas et al. 2023 Miguel-Rojas et al. https://creativecommons.org/licenses/by/4.0/ This content is distributed under the terms of the Creative Commons Attribution 4.0 International license.

### Changes in gene expression during appressorium development.

In M. oryzae, gene enrichment analysis highlighted stage 4 as having the highest category diversity (Table S2B). Conidia undergo autophagy at stages 1 to 3 in preparation for appressorium formation, which greatly decreases gene expression. Stage 4 gene expression profiles were analyzed to determine gene functions associated with initial growth in plant tissue. We identified the 100 top upregulated genes at stage 4 (Table S3B). Among these genes, 62% showed secretion signal peptides, which could include secreted fungal effectors (65). In addition, the majority of the 100 genes (69%) encoded hypothetical proteins with unknown function, indicating the need for further gene functional characterization. The remaining 31% of well-annotated genes mainly encode CAZyme degradative enzymes, secondary metabolite enzymes, and transporters, suggesting activity in the degradation of the plant cell wall, defense against antifungal compounds produced by the plant, and transport of the acquired nutrients from the host.

During initiation of the infection process, pathogenic fungi are exposed to a nutrient-limited environment. Therefore, metabolism directed at harnessing energy plays a key role for success in colonization. In F. graminearum, the expression patterns of 176 genes encoding enzymes belonging to energy-harnessing metabolic pathways, including the glyoxylate cycle, tricarboxylic acid (TCA) cycle, and fatty acid oxidation, indicated that pathways that produce acetyl-coenzyme A (acetyl-CoA) are active during the infection process (Table S4A). The key enzymes of the glyoxylate pathway (isocitrate lyase and malate synthase) were strongly induced at stage 3, leading to appressorium formation.

10.1128/mbio.02442-22.7 TABLE S4 (A) Analysis of metabolic pathways during F. graminearum infection on host. (B) Analysis of pathways that directly use or produce acetyl-CoA in M. oryzae on host. (C) Analysis of genes encoding secreted CAZymes on host in F. graminearum. (D) Analysis of genes encoding secreted CAZymes on host in M. oryzae. (E) Analysis of genes encoding chitin deacetylases (CDAs) in F. graminearum and M. oryzae on host. (F) Analysis of genes encoding putative effector proteins with known protein domains and unknown domains on host in F. graminearum. (G) Analysis of genes encoding identified effector proteins on host in M. oryzae. Download Table S4, XLSX file, 0.6 MB. Copyright © 2023 Miguel-Rojas et al. 2023 Miguel-Rojas et al. https://creativecommons.org/licenses/by/4.0/ This content is distributed under the terms of the Creative Commons Attribution 4.0 International license.

In M. oryzae, acetyl-CoA biosynthesis and metabolism also play crucial roles in appressorium function during host infection (66–70). We examined 47 genes encoding enzymes belonging to different pathways that produce or utilize acetyl-CoA (Table S4B). Previous studies reported that acetyl-CoA has an essential role in development of functional appressoria and is mainly produced from beta-oxidation of fatty acids, transported out of the peroxisome by carnitine acetyltransferase, and used for the glyoxylate cycle (43, 69). Our results support these findings that together these two pathways allow carbon units to be used for glycerol biosynthesis via gluconeogenesis (67), resulting in the availability of glycerol, which is essential for turgor pressure buildup during the infection process.

The genome of F. graminearum contains 587 annotated genes encoding CAZymes. Expression analysis was performed for five important families: polysaccharide lyases, glycosyl transferases, proteins harboring carbohydrate-binding modules, carbohydrate esterases, and glycoside hydrolases (Table S4C). Among the CAZymes, the cell wall-degrading enzymes were the most abundant and have essential roles in penetration, invasion, pathogenicity, and virulence (71–75). Our data revealed that a high number of genes encoding cell wall-degrading enzymes were strongly upregulated in conidia germinating on the host, with the highest expression overall at stages 3 and 4.

M. oryzae also relies on the secretion of a large repertoire of enzymes to break down the plant tissue. Expression data identified 188 active genes encoding CAZymes (Table S4D). Of these, 119 (63%) were significantly upregulated during conidial germination on the host versus in Bird medium and mainly at stage 4. The classes of enzymes included many involved in the degradation of the plant cell wall. Furthermore, genes involved in fungal cell wall modification, including chitinases, glucanases, mannosidases, and beta-hexosaminidases, were also differentially expressed.

Chitin deacetylases (CDAs) catalyze the removal of acetyl groups from chitin, one of the main polysaccharide components of the wall, forming chitosan, which acts with chitin to provide structural support for fungal cells (76) and has been shown to prevent ligand-triggered immunity during plant infection (77). Our results showed that as the germination process progressed from early to late stages, 6 of 12 CDA genes present in M. oryzae were upregulated and are likely involved in the adhesion of the fungus to the surface (Table S4E). In F. graminearum, six of nine putative CDA genes were mainly expressed on the host at stage 4. For F. graminearum, adhesion is most important and most visible at the formation of penetration structures (7, 16, 34).

Mentges et al. (7) analyzed the secreted fungal putative effector (PE) proteins during palea infection and specifically in infection cushions in wheat, defining PE as secreted proteins without transmembrane domains and having 1,000 amino acids as a maximum size. By comparison, we analyzed the expression of putative effectors in our transcriptomic analysis (Table S4F). Sixty-six undescribed effectors were identified with no predicted domains but were classified according to their taxonomic specificity based on Mentges et al. (7). Specifically, 48 undescribed effectors were upregulated at stage 4, and 15 of these effectors were shared with those expressed in infection cushions. In addition, 17 of 20 previously described effectors were upregulated across the spore germination process on the host during appressorium formation, sharing six of eight with those upregulated in infection cushions.

Expression of 42 previously described effectors in M. oryzae was analyzed in our transcriptomic data set (Table S4G). In addition, gene expression analysis was performed on another 33 secreted effector proteins previously described by Chen et al. (78) (Table S4G).

### Expression of genes for synthesis of secondary metabolites (SM), autophagy-related processes, and hydrophobins.

**FIG 6:**
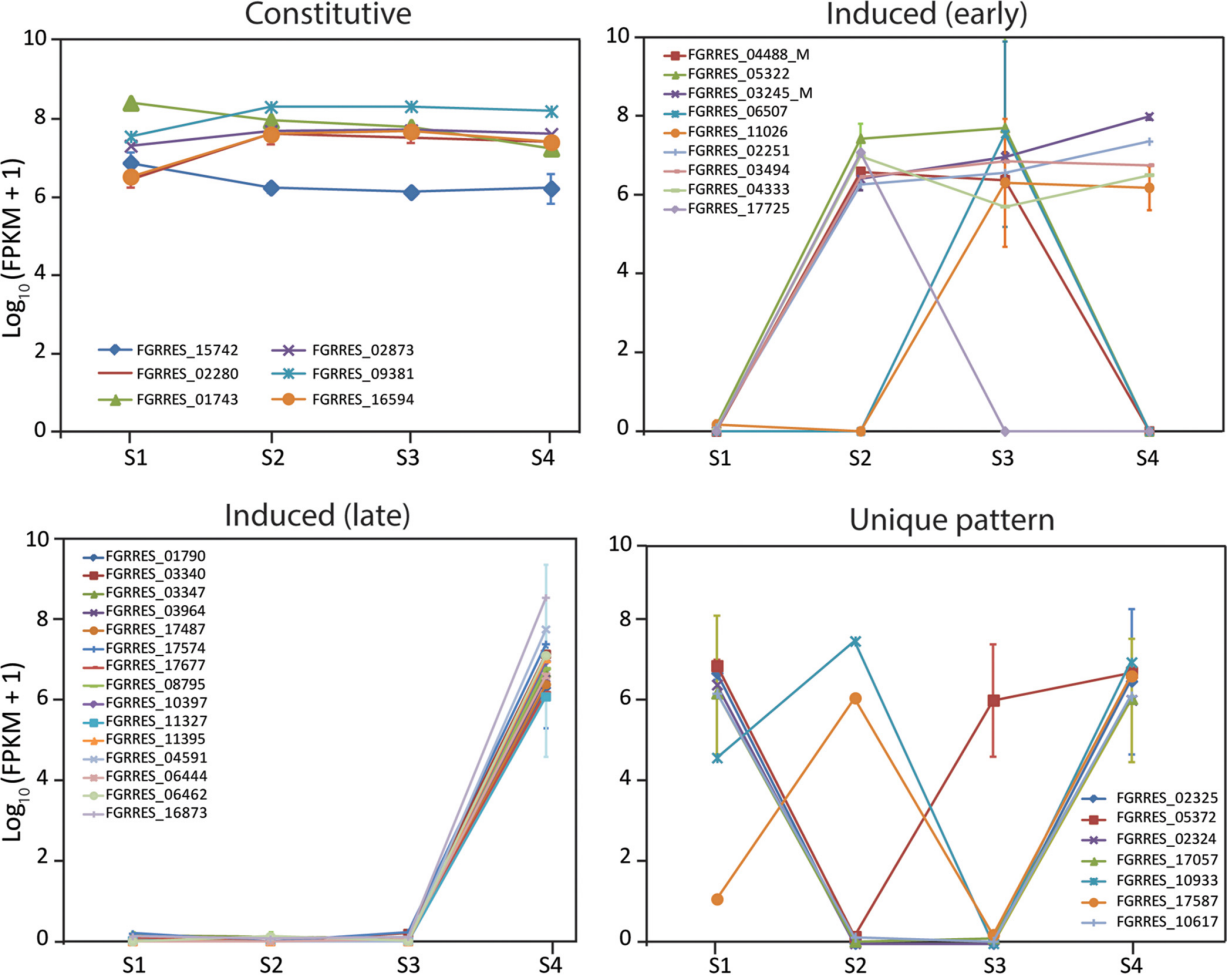
Expression profiles of secondary metabolite genes, presented as log_10_ (fragments per kilobase per million [FPKM] + 1), during the stages of spore germination in F. graminearum on barley sheaths. Genes with similar expression patterns are grouped, with the final group composed of genes that have unique expression patterns.

**FIG 7:**
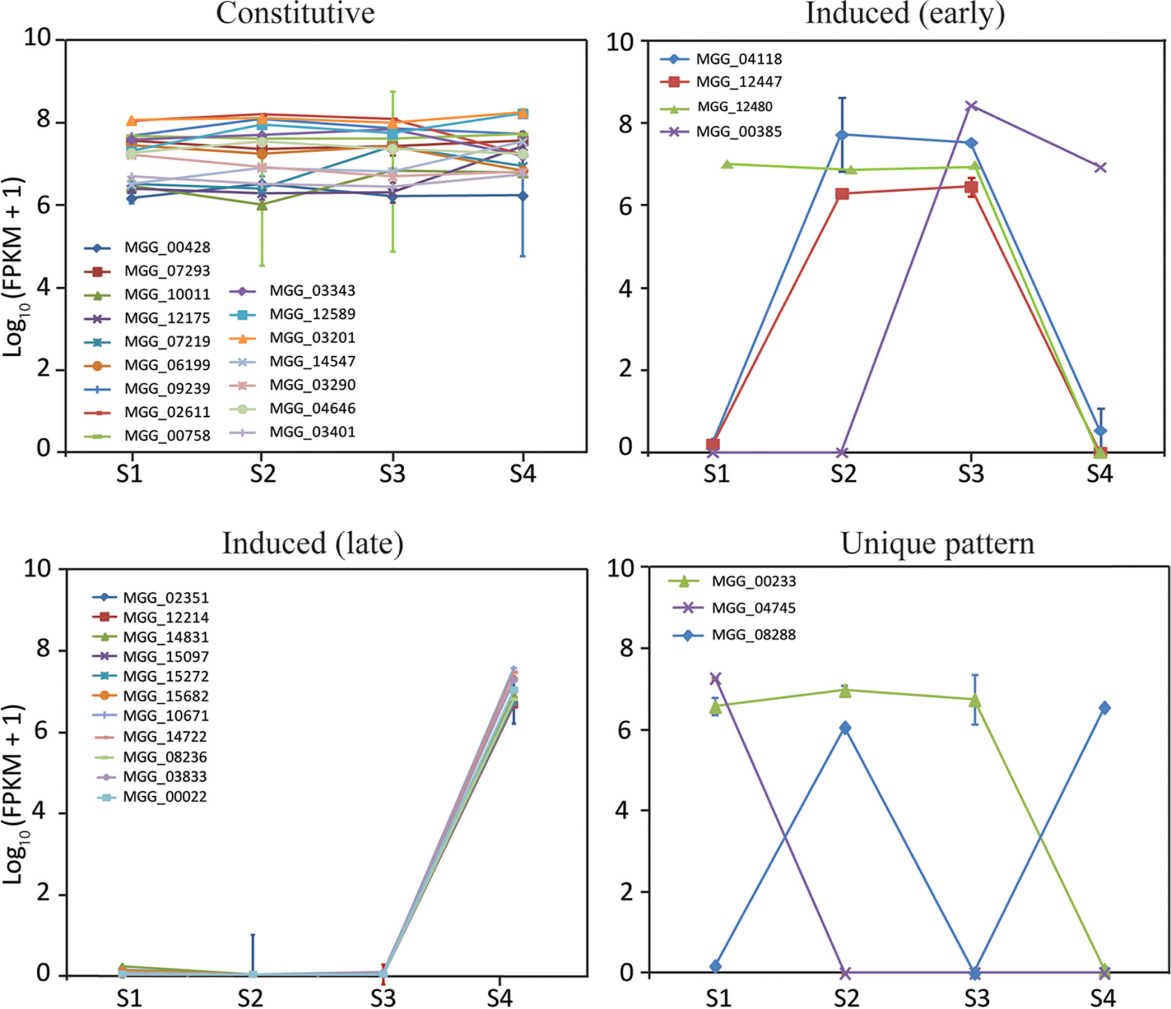
Expression profiles of secondary metabolite genes, presented as log_10_ (FPKM + 1), during the Stages of spore germination in M. oryzae on barley sheaths. Genes with similar expression patterns are grouped, with the final group composed of genes that have unique expression patterns.

Transcriptomic data were used to determine and compare the expression profiles of genes involved in synthesis of SMs, autophagy-related enzymes, and hydrophobins during spore germination on the host versus in Bird medium in both species. Fifty-five SM genes in F. graminearum and 50 SM genes in M. oryzae encoding the major SM biosynthetic genes (nonribosomal peptide synthases, terpene synthases, and polyketide synthases) were identified (Tables S5A and B). Of the genes in F. graminearum, at stage 4, where appressorium development occurs, 27 SM genes were highly upregulated as indicated by their log_2_ fold change values. In M. oryzae, 20 genes were upregulated predominantly at stage 4. SM genes that are orthologous between the two species are indicated in Table S5C. Expression levels of polyketide synthase (PKS), terpene synthase (TS), and nonribosomal peptide synthase (NRPS) genes from each species on the host and in medium were compared and separated into common expression categories: constitutive, induced (early), induced (late), and no common pattern (Fig. 6 and 7). Interestingly, in F. graminearum, the majority of SM genes were induced late (stage 4; 16 of 38 genes), whereas in M. oryzae the majority of genes were expressed constitutively (16 of 34 genes).

10.1128/mbio.02442-22.8 TABLE S5 (A) Analysis of genes encoding secondary metabolites in F. graminearum on host. (B) Analysis of genes encoding secondary metabolites in M. oryzae on host. (C) SM genes in F. graminearum and their orthologs in M. oryzae. (D) Expression profiles of TRI genes associated with DON synthesis presented as log_10_ (FPKM + 1) on medium and on the host in F. graminearum. Download Table S5, XLSX file, 0.3 MB. Copyright © 2023 Miguel-Rojas et al. 2023 Miguel-Rojas et al. https://creativecommons.org/licenses/by/4.0/ This content is distributed under the terms of the Creative Commons Attribution 4.0 International license.

Expression of the Tri genes for deoxynivalenol (trichothecene) biosynthesis in F. graminearum were also revealed by transcriptomic analysis. Expression assays on wheat sheaths showed that the Tri genes *Tri5*, *Tri6*, *Tri8*, *Tri9*, and *Tri14* lacked significant expression during the stages of conidial germination and plant penetration, whereas *Tri1*, *Tri3*, *Tri4*, *Tri7B*, *Tri10*, *Tri11*, and *Tri12* showed significant expression only at stage 4, and *Tri101* showed significant expression only at stage 2 (Fig. S2 and Table S5D). All Tri genes examined lacked significant expression on Bird medium at any stage. In the laser-dissection data, expression of Tri genes was not apparent, and, overall, expression of genes for secondary metabolites were not identified in the data. However, the quality and quantity of reads were not as robust as in our other experiments.

10.1128/mbio.02442-22.2 FIG S2 Expression profiles of TRI genes in F. graminearum on host presented as log_10_ (FPKM + 1). Download FIG S2, TIF file, 0.5 MB. Copyright © 2023 Miguel-Rojas et al. 2023 Miguel-Rojas et al. https://creativecommons.org/licenses/by/4.0/ This content is distributed under the terms of the Creative Commons Attribution 4.0 International license.

In F. graminearum, eight ATG genes, *ATG5*, *6*, *12*, *13*, *14*,*18*, *22*, and *24*, exhibited unique patterns of expression across the time course on the host versus on Bird medium, including stages where they were at very low expression levels (Fig. S3 and Table S6). However, only *ATG12* and *ATG13* were expressed on the host during plant penetration (stage 4). In M. oryzae, expression of ATG genes was uniformly high both on Bird medium and on the host (Fig. S3 and Table S6).

10.1128/mbio.02442-22.3 FIG S3 Expression profiles of autophagy-related (ATG) genes presented as log_10_ (FPKM + 1) on medium and on the host. Download FIG S3, TIF file, 2.2 MB. Copyright © 2023 Miguel-Rojas et al. 2023 Miguel-Rojas et al. https://creativecommons.org/licenses/by/4.0/ This content is distributed under the terms of the Creative Commons Attribution 4.0 International license.

10.1128/mbio.02442-22.9 TABLE S6 Expression profiles of autophagy-related (ATG) genes presented as log_10_ (FPKM + 1) on medium and on the host in F. graminearum and M. oryzae. Download Table S6, XLSX file, 0.1 MB. Copyright © 2023 Miguel-Rojas et al. 2023 Miguel-Rojas et al. https://creativecommons.org/licenses/by/4.0/ This content is distributed under the terms of the Creative Commons Attribution 4.0 International license.

Fungal hydrophobins are secreted hydrophobic proteins that support the formation of aerial structures. Hydrophobins also mediate the attachment of the hyphae to hydrophobic surfaces and are essential for the initial steps of fungal pathogenesis when the fungus must attach to the host before penetration (79, 80). The genome of F. graminearum contains five genes encoding hydrophobins (*FGHyd1* to *FGHyd5*) (81, 82). Analysis of gene expression shows that *Hyd1* to *Hyd3* were expressed at high levels at later stages (3 and 4) of conidial germination on the host (Table S7). In M. oryzae, expression of 12 hydrophobin proteins have been analyzed (Table S7). Our results are similar to previous studies (43).

10.1128/mbio.02442-22.10 TABLE S7 Analysis of genes encoding hydrophobins in F. graminearum and M. oryzae on host. Download Table S7, XLSX file, 0.1 MB. Copyright © 2023 Miguel-Rojas et al. 2023 Miguel-Rojas et al. https://creativecommons.org/licenses/by/4.0/ This content is distributed under the terms of the Creative Commons Attribution 4.0 International license.

## DISCUSSION

Here, we present a comparative RNA-sequencing (RNA-seq) approach to assess the underlying genetic and physiological divergence in spore germination and very early plant-fungal interactions in two well-studied pathogens with distinct infection strategies. Four sequential stages of development following spore localization on the plant surface, from spore swelling to appressorium formation, were sampled for each species on a defined medium (saprotrophic conditions) and on barley sheaths (pathogenic conditions), and transcriptomic analyses were performed.

Gene expression in the prepenetration stages in both species and under both conditions was similar. In contrast, gene expression in the final stage was strongly influenced by the environment. Laser-dissection microscopy was used to perform detailed transcriptomics of initial infection points by F. graminearum. Expression of the trichothecene genes involved in biosynthesis of deoxynivalenol by F. graminearum implies that toxisomes are not fully functional until after penetration and indicates that deoxynivalenol is not essential for penetration under our conditions. Expression of autophagy genes that were distinct to each species implies differential application of autophagy in the infection process. These results present the initiation of the pathogenic cycle in greater detail than previous studies.

In this study, conidia of F. graminearum germinating on the host demonstrated marked increases in expression of a subset of genes associated with DON biosynthesis (*Tri1, Tri3, Tri4, Tri7B, Tri10, Tri11, and Tri12*) between stages 3 and 4. Toxisomes, which harbor the proteins for DON biosynthesis, are derived from the endoplasmic reticulum (83, 84). Tri1, Tri4, and Tri11 have been shown to be integral to toxisome membranes, whereas other Tri biosynthetic proteins are found in the cytosol or other cellular membranes (85). The expression of these three genes just as the fungus is penetrating the host together with the localization of their proteins on the endoplasmic reticulum (ER) membrane determined previously (85) suggests that this time point marks the initiation of toxisome formation. Transcripts of the trichodiene synthase gene (*Tri5*), encoding the major enzyme for trichothecene biosynthesis, were not detected during the stages we examined on barley sheaths (up to 48 h), despite evidence that DON biosynthetic genes have highly coordinated expression, specifically with *Tri5* (86, 87). However, the data from previously published experiments showing coordinated expression were acquired at later time points than we studied here, or data from several time points were combined so that it is not possible to distinguish expression in early and later time points (7). For example, in a study of Tri gene expression in isolates of F. graminearum with different toxin profiles on a susceptible wheat cultivar, *Tri5* transcripts were present at 2 dpi (when the sampling began) and peaked at 4 dpi, and *Tri4* transcript production was present at 2 dpi and peaked at 3 to 4 dpi (88). Tri5 has been found to attach to Tri11 and form bridges by homodimerization, contributing to the association of ER membranes in a stack, characteristic of the toxisomes (84). We do not know the longevity of the proteins or mRNA within the cells, which would be important for evaluating timing of protein activity. However, our data do strongly suggest that there is processive expression of genes for DON biosynthesis during toxisome development, which had not been previously shown. Our data indicate that, under our conditions, the fungus is inside the host when the toxisomes are formed, that DON production is initiated after penetration, and, furthermore, that the presence of DON is not important in initiating fungal ingress. This is further evidence that early DON production is likely absent or in very small amounts during germination and appressorium formation under the conditions we studied. These findings provide evidence that the ER in F. graminearum forms the basic membranes for toxisomes in the initial stages of penetration and has them ready to mature quickly once the host has been penetrated.

Tri6 and Tri10 are transcription factors that have been shown to regulate overlapping genes involved in trichothecene biosynthesis, and there is evidence that Tri6 has broader function in this regard (89, 90). The data presented here suggest that Tri10 may be active very early in the process of pathogen ingress and may regulate expression of Tri genes integral to the toxisome membrane. *Tri101* was an outlier among the Tri genes in our expression analysis on the host, exhibiting expression at stage 2. The *Tri101* protein catalyzes the acetylation of several trichothecene mycotoxins at the C3 position, which has been suggested to reduce toxicity of the mycotoxin and thus protect the mycelia before secretion of the toxin, at which time the acetyl group is removed (91–93). Early expression of this gene was surprising but may suggest that the consequences of not having it present when the mycotoxin is synthesized are severe. A study by Amarasinghe and Fernando (88) showed that *Tri101* expression was consistent for a longer time on the susceptible cultivar, again supporting the importance of protection of the fungus from mycotoxin toxicity. It would be interesting to compare the expression of this gene in cultivars with different types of resistance to determine if *Tri101* is more frequently kept on when the mycotoxin is produced for a longer time.

The majority of SM genes spike in expression at stage 4 in F. graminearum, whereas in M. oryzae, the majority of SM genes are expressed constitutively through all stages. These results reflect the very different uses of SMs in infection between the two fungi. Whereas F. graminearum may exhibit greater use of SMs to prepare the infection court, M. oryzae uses predominantly bulk pressure for ingress, which is dependent on melanin biosynthesis (94) but may not depend as much on assistance from other chemistries. LaeA, a global regulator of secondary metabolism in many fungi (95–97), has no discernible role in the pathogenicity of M. oryzae (98) but profoundly affects production of DON and zearalenone in F. graminearum (99). However, while SMs are known to play important roles during pathogenicity in many fungi (100–102), less comprehensive analyses have been done for SMs in M. oryzae (103).

A comparison of transcriptional profiling of genes involved in autophagy (ATG) between M. oryzae and F. graminearum shows a strikingly divergent expression pattern on the plant during spore germination and penetration. The ATG genes in M. oryzae exhibit nearly uniform robust expression during all four stages studied compared to F. graminearum, in which a few of the individual genes are expressed in unique patterns. Previous studies of ATG genes during pathogenicity in M. oryzae showed that autophagy plays a role in infection-related processes of conidia and appressoria (36, 104, 105). Specifically, as conidia germinate and form appressoria, autophagy within the conidium allows cellular resources to migrate to germ tubes and then to developing appressoria. Previous studies in F. graminearum demonstrate that ATG genes contribute to several activities that contribute to pathogenicity and colonization: hyphal colonization (shown on agar medium), conidiation, lipid catabolism, and DON biosynthesis (106–108). Germ tubes of F. graminearum establish a more robust surface presence than do germ tubes of M. oryzae, as runner hyphae grow for some distance to locate trichomes, silica cells, and other points of entry (7, 16). The cells of the expanding hyphae do not undergo autophagy behind the growing front, and germlings on the plant surface are likely to continue to take up nutrients as they grow and increase biomass. Thus, ATG genes have multiple purposes and are expressed at different times in F. graminearum and M. oryzae, and the impacts of these processes are not completely understood particularly in F. graminearum.

Laser-dissection analysis was used to probe gene expression in F. graminearum during ingress into the cells of the epidermis (stage 5), primarily the trichomes. Laser dissection followed by transcriptomics has been previously reported for surface tissues of infected wheat at 4 to 5 dpi (7), where substantially developed infection hyphae occur, and expression of Tri genes and virulence factors were identified. Upregulated genes in the infection cushions identified in that study overlap with those upregulated in our study. Our RNA-seq results from the laser-dissection analysis clearly support catalytic degradation of the plant tissue once the fungus penetrates the host (stage 5), followed by rapid intracellular growth of the fungus in the host. Development and spread of symptoms after a very brief symptomless period is characteristic of head blight development (6, 109). Both fungi use the plasmodesmata to move from cell to cell (16, 109, 110), with F. graminearum having a very short biotrophic stage limited to the movement through the first few layers of the chlorenchyma (16, 109, 111), whereas M. oryzae exhibits an extended, highly studied biotrophic phase (35, 36, 112, 113).

The target of rapamycin (TOR) signaling pathway strongly inhibits development of appressoria in M. oryzae and is controlled, at least in part, by cellular glucose levels. During spore germination on the plant surface, an inductive, nutrient-limited hydrophobic surface, the TOR signaling pathway is off, arresting the cell cycle. This arrest in G_2_ induces autophagy and appressorium formation, important processes for infection (114, 115), as observed here at stage 4. Glucose treatment activates TOR, and the cell cycle resumes (115, 116). On Bird medium, a noninductive hydrophilic surface containing glucose, spore germination results in constitutive expression of TOR, enabling multiple rounds of mitosis and long germ tubes as observed at stage 4. In F. graminearum, TOR signaling affects vegetative grown and conidial germination and functions in mycotoxin production, virulence, and sexual reproduction (117–119); thus, its role appears more diffuse and multifaceted than has been currently documented for M. oryzae.

Conidia of F. graminearum and M. oryzae germinated on the host versus in medium during stage 4 revealed a noticeably greater number of genes in both fungi that are upregulated on the host. Furthermore, in comparison to other stages, approximately half of these genes have no known function, a striking result considering the history of concentrated research focused on these two pathogens (36, 120–124). The greater number of upregulated genes throughout the germination and penetration processes in F. graminearum than in M. oryzae may reflect the larger and more complex structure formed by F. graminearum. F. graminearum enters the plant directly in association with trichomes (16, 125, 126), but penetration follows the formation of hyphopodia, and as disease progresses, single recognizable hyphopodia develop into infection cushions comprised of many appressoria beneath layers of hyphae (7, 16, 34, 111). In addition, gene expression may be more limited in M. oryzae, as germinating spores undergo autophagic fungal cell death as they prepare for development of a single specialized appressorium at the end of a short germ tube (24–28, 58). These results demonstrate vast transcriptional reprogramming associated with these stages of host infection and reflect our general lack of understanding about the diversity of genes underpinning these transitions among fungi in this important aspect of the disease cycle.

The most striking contrast between the processes of ingress into hosts by these two plant pathogens are the melanized appressoria produced by M. oryzae versus hyaline hyphopodia produced by F. graminearum. The former strategy allows M. oryzae to penetrate the plant surface immediately, and, in support of this, their spores have a sticky tip to attach to leaves they land on (127). In contrast, the formation of hyphopodia after homing in on a trichome or finding a crack in the host epidermis, as in F. graminearum, takes more extensive growth. Furthermore, M. oryzae infects mainly leaves, whereas F. graminearum infects flowers and stalks but is not a leaf pathogen. Differences in target tissue may have resulted in the different approaches. F. graminearum takes advantage of multiple entry points, including breaches in the florets due to senescence of flower parts and the ability to take advantage of trichomes and other silica cells to breach the epidermis (16).

Previously, we paired transcriptomic analysis of diverse fungal species undergoing sexual sporulation with ancestral reconstruction analysis to identify genes with the largest evolved increases in gene expression across development. Phenotypic analyses of mutants of these genes resulted in a highly successful means of finding genes with important roles in sexual development (51, 52). This process is now being performed for spore germination in M. oryzae and F. graminearum together with several other fungal genera representing diverse lineages. Shedding light on the basis of diversity in the infection process will result in development of more specific control strategies. The threat of both of these pathogens causing a major disease outbreak affecting food security for millions of people is ever present (127). To discover effective disease prevention and treatment options, it is imperative to identify components of the transcriptional program underlying initial stages of disease.

## MATERIALS AND METHODS

### Fungal strains and culture conditions.

The wild-type F. graminearum strain PH-1 (FGSG9075, NRRL31084), which has been previously described (128), was maintained as conidial stocks and colonized pieces of V8 agar in sterile 35% glycerol at −80°C. Lyophilized stocks of M. oryzae strain 70-15 (FGSC8958) on colonized filter paper were stored at −80°C and at room temperature (RT). The genomes of both strains have been sequenced (120–122).

### Developmental stages.

Asexual development was monitored from germination to hyphal branching on 2% agar Bird medium (129), a defined minimal medium, and to appressorium formation *in planta*. Bird medium was developed by R. Metzenberg to reduce issues occurring with use of some minimal defined media formulations, resulting in effects on physiology and thus gene expression, particularly for studies of spore germination (129). This medium was chosen for an ongoing comparative study of spore germination in several genera of fungi, of which this study is a part (16, 48–53). In *M. grisea*, germ tube branching is not the usual pattern of early development leading to pathogenicity. However, the purpose of germination on Bird medium, together with germination on the host, is to identify genes involved in infection of barley that have little to do with germination in general, and the medium is sufficient for this purpose. Furthermore, keeping the common garden aspect for the great variety of fungi we have studied and are studying is essential (16, 48–53). For convenience, selected asexual stages were labeled by the approximate time at which the designated stages occurred (Fig. 1). The selected stages were as follows: stage 1, fresh conidia; stage 2, polar growth; stage 3, doubling of long axis; stage 4, first hyphal branching or appressorium formation on Bird medium or on the host, respectively. Germination was performed for both species with fresh conidia from 5-day cultures shaking in carboxymethylcellulose medium (130) for F. graminearum and 10-day cultures on solidified defined complete medium (131) for M. oryzae. Conidia for each species were harvested by filtration through three layers of Miracloth (Millipore, Billerica, MA) and, before use, washed three times with sterile deionized distilled water containing Tween 20 (0.1%). Samples for each stage were collected when approximately 80% of the active spores showed the morphological characteristics corresponding to that specific stage. For generation of samples on Bird medium, 1 × 10^5^ total conidia were inoculated onto medium covered with autoclaved cellophane in petri dishes (diameter of 60 mm; Falcon) and incubated at 24°C under continuous light. Cellophane membranes bearing fungal tissue were collected at specific postinoculation stages (Fig. 1). Approximately 250 membranes from each stage were pooled for each experiment. Three independent experiments were performed per species, each representing a biological replicate.

For host inoculation, barley (*Hordeum vulgare* L.) cultivars Stander and Golden Promise were sown in Suremix medium (Michigan Grower Products, Inc., Galesburg, MI) and grown in the greenhouse under supplemental lighting with a 16-h day at approximately 22°C. Three-week-old seedlings were used for inoculation of F. graminearum (cultivar Stander) and M. oryzae (cultivar Golden Promise). Conidia were adjusted to 2.5 × 10^5^/mL in Tween 20 (0.1%). Leaf sheaths from intermediate-aged leaves from the seedlings were cut into sections approximately 5 cm long and hand-trimmed along the midvein to expose the epidermal layer. Each strip was inoculated with five 10-μL droplets of conidial suspension placed along the length of the epidermis. Inoculated sheaths were placed horizontally in a square culture dish (120 mm × 120 mm) with each end on a glass slide, and the center area was suspended above wet filter paper to maintain high humidity. This setup discouraged growth of hyphae across the slides. Plates were sealed to retain moisture. Inoculated leaf sheaths were incubated at 24°C under continuous light and harvested at the prespecified stages following inoculation (Fig. 1). Twenty leaf sheaths (each from a different plant) were harvested for each stage and pooled for each experiment. Three independent experiments were performed, each representing a biological replicate. Harvested samples were flash-frozen in liquid nitrogen and stored at −80°C until processed.

### Detached floret infection assay.

Detached floret infection assays similar to those previously described (16, 132) were performed as follows. Heads of flowering barley were selected following the initial opening of the flag leaf sheath to reveal the first spikelet (Zadoks stage 47 to 51) (133). For each assay, the lowest 3 to 5 healthy spikelets from at least five heads were removed. The individual florets were then isolated, glumes were excised, and awns were trimmed to ~3 to 4 mm above the lemmas. Florets were placed in a screw-cap tube containing 2.5 × 10^5^ conidia in suspension. The tube was slowly inverted for 60 s to coat the florets. Florets were then removed from the suspension with forceps, touched against the side of the tube to drain excess fluid, and placed with the bases down into fresh 0.9% water agar in 100 mm × 20 mm petri dishes. The petri dishes were incubated under continuous white fluorescent light at room temperature (22 to 24°C). Florets were collected from 4 to 6 dpi. Two independent biological replicates were generated.

### Laser-capture microscopy.

RNase-free reagents and labware were used during all steps described in this section. Methods for isolation of high-quality RNA through microdissection were modified from Hong et al. (134). RNase-free 0.1% toluidine blue and Safranin O aqueous solutions were generated by baking the powdered dyes in bottles for 6 to 8 h at ≥180°C before adding RNase-free water. Inoculated florets were removed from culture dishes and placed into 50-mL conical tubes, and prechilled Carnoy’s solution (6:3:1, 95% ethanol:chloroform:glacial acetic acid) was added. The samples were incubated on ice for 2 h with 3 to 4 gentle inversions every 30 min. To clear the plant tissue, the samples were incubated in fresh Carnoy’s solution at 4°C, exchanging the solution for fresh every 18 to 24 h until the fresh solution remained visually colorless (generally 2 to 4 days). The samples were either moved directly to staining or, after a final change to fresh Carnoy’s solution, were stored at −80°C. To stain the florets, the Carnoy’s solution was removed, and florets were washed twice for 5 min each with 1× phosphate-buffered saline (PBS; pH 7.4). The intact florets, inoculated and uninoculated, were stained for lignin, and cells wall components were stained in 0.1% toluidine blue (135) at 4°C for 18 to 24 h, washed twice with 1× PBS (pH 7.4), and counterstained with 0.1% safranin O at 4°C for 2 to 4 h, causing the lignified walls to stain red (136, 137). The 1× PBS (pH 7.4) washes were repeated a final time. Lemmas and paleae were removed from florets and placed in the same orientation into cryogenic molds inside a sealable container and layered with optimal cutting temperature (OCT) medium (Sakura Finetek USA, Maumee, OH). To facilitate layering, the container with the cryogenic molds was sealed and placed at −80°C for 5 min every 2 to 3 layers. Zeiss MembraneSlide 1.0 PEN or nuclease-free MembraneSlide NF 1.0 PEN slides (ZEISS Group, Oberkochen, Germany) were irradiated with 254-nm UV light for 30 min to increase the adhesion of sections to the membrane. Slides were placed in staining dishes and wrapped with aluminum foil. Dishes containing MembraneSlides were baked at ≥180°C for 6 to 8 h and cooled before being stored until use at room temperature with the unbaked dishes containing MembraneSlide NF slides.

Frozen samples were sectioned at −20°C to thicknesses ranging from 5 to 50 μm with a Leica CM 1850 cryostat (Wetzlar, Germany). All removable parts from the cryostat were warmed to >0°C, treated with RNase Away, rinsed with water, and dried before being reinstalled into the cryostat. Nonremovable parts were wiped with absolute ethanol. Paint brushes used to manipulate sections onto the slides were treated with RNase Away for 1 min, rinsed twice with water, and dried using a Kimwipe (Kimberly Clark, Irving, TX) to wick away water, followed by air drying for 2 min at RT. Slides were kept at RT between the mounting of each section so that the OCT medium melted on contact, helping sections stick to the membrane. Filled slides were placed back into the staining dish at RT while sectioning continued. Once sectioning was completed, slides were either used directly for laser-capture microscopy or were stored at −80°C inside the aluminum foil-wrapped staining dishes until processed.

A Carl Zeiss MicroImaging PALM MicroBeam laser-capture microscope, release 4.3SP2, operated with PALMRobo software, version 4.3.2.13, equipped with a RoboMover automated stage was used to observe, image, and capture regions of interest (ROIs) from sections. Images were captured using a Zeiss AxioCam ICc 1 camera. Catapulted ROIs were collected using Zeiss AdhesiveCap clear or AdhesiveCap opaque (200 μL). AxioVision software version 4.8.1.0 was embedded in the PALMRobo software.

Infected cells were predominantly trichomes and epidermal and mesophyll cells. Captured cells that were not trichomes were on the surface or within two cell layers of the surface, with a 3:1 proportion of surface:nonsurface cells captured. Most captures consisted of single cells, but where adjacent cells appeared to be independently infected, up to three adjacent cells were captured. Larger lesions, where the outer epidermal cells were no longer present and the cell walls were degraded were avoided. Areas where the fungus was moving extracellularly or through plant walls were also avoided.

### RNA isolation, RNA-seq library construction, and Illumina sequencing.

Colonized cellophane membranes and infected leaf sheaths were ground in liquid nitrogen, and total RNA was extracted from these two tissues using TRIzol reagent (Thermo Fisher Scientific, Waltham, MA) and the Qiagen RNeasy plant minikit (Qiagen, Germany) according to manufacturer’s instructions, respectively. Total RNA from laser-capture samples of detached florets was extracted immediately after microdissection using the ARCTURUS PicoPure RNA isolation kit (ThermoFisher, Waltham, MA). Part I of the manufacturer’s protocol was followed, and samples were stored at −80°C until ≥500 ROIs had been captured. At that point, part II of the manufacturer’s protocol was followed to isolate RNA. RNA samples were concentrated using RNA clean and concentrator (Zymo Research Corp., Irvine, CA) after genomic digestion with the RNase-free DNase set (Qiagen, Hilden, Germany). RNA was eluted in RNase-free water and checked for integrity and quantity on an Agilent 2100 Bioanalyzer according to manufacturer’s instructions. RNA was prepared from two laser-capture samples or from three cellophane membranes plus infected leaf-sheath samples as biological replicates prepared as independent RNA libraries. Total RNA (2 μg) was used to construct strand-specific cDNA libraries from poly(A)-captured RNAs using the Kapa stranded RNA-seq library preparation kit (Kapa Biosystems, Wilmington, MA). Strand-specific cDNA libraries were sequenced on the Illumina HiSeq 2500 platform (Illumina, Inc., San Diego, CA) at Michigan State University Research Technology Support Facility (https://rtsf.natsci.msu.edu/genomics).

### Transcriptomic analysis.

The quality of raw reads (single end, 50 bp) was assessed with the FastQC program (v0.11.3; www.bioinformatics.babraham.ac.uk/projects/fastqc). Poor-quality reads, adapters, and homopolymers were removed using the ngsShoRT program (v2.2) (138), with option arguments “lqr_5adpt_tera,” “-5a_f n-1,” “-5a_fmi 40,” “-5a_mp 95,” and “–rmHP.” Filtered reads were then mapped to the reference genome using the HISAT2 program (v2.0.4) (139). BAM files generated from HISAT2 output were sorted using SAMtools (v1.3.1) (140). StringTie (v1.3.0) (141) was used to generate a genome-guided transcriptome assembly. The reference genomes were downloaded from https://fungi.ensembl.org/index.html (F. graminearum RR.34 version and M. oryzae MG8.34 version). “FGRRES” is the ID used to identify the F. graminearum genes in the genome version RR.34 used in this study (122).

### Differential gene expression and functional enrichment analysis.

Read counts for gene loci were calculated using the htseq-count program (v0.6.1) (142). Blast2GO suite (143) was used for downstream analysis. Gene expression levels in counts per million (CPM) values were normalized by effective library size, as estimated by the trimmed mean of M values (57). Only genes with CPM values greater than 1 in at least 3 samples and differentially expressed (DE) genes showing a false discovery rate (FDR) of 5% were kept for further analysis. Functional enrichment analysis for DE genes was performed using FungiFun2 (144). To assess enrichment of GO terms, the hypergeometric distribution and Benjamini-Hochberg methods were used to calculate the FDR-corrected *P* value. The statistical significance of overrepresentation of gene groups in functional categories (FunCat analysis) relative to the whole genome was quantified by calculating *P* values via the hypergeometric distribution using FungiFun2 (https://elbe.hki-jena.de/fungifun/). To achieve significance, we required both an exact *P* value of 0.01 and an adjusted *P* value of 0.05.

### Secondary metabolites, autophagy-related genes, and hydrophobin genes.

Transcriptomic data were used to determine the expression profile of secondary metabolites (SMs), autophagy-related (ATG) genes, and hydrophobins during the spore germination profiles in both species studied. Genes encoding polyketide synthases (PKSs), terpene synthases (TSs), and nonribosomal peptide synthases (NRPSs) were identified through SM clusters shown at https://mycocosm.jgi.doe.gov/Fusgr1/Fusgr1.home.html and through GenBank annotations as well as annotations reported by Trallamazza et al. (145) and Villafana, Ramdass, and Rampersad (146). ATG genes were identified through annotations reported by Lv et al. (106). Orthologs between the two species were identified by reciprocal BLASTp analysis through NCBI (https://blast.ncbi.nlm.nih.gov/Blast.cgi?PAGE=Proteins) between each gene against the other species using predicted protein sequence comparisons with a cutoff protein sequence of the best hit. The best hit was based on the lowest *e* value, highest query cover, and maximum score, taking into consideration predicted domains, resulting in ortholog predictions and comparisons of expression (Table S5C in the supplemental material). Genes encoding hydrophobins were identified through GenBank annotations as well as annotations reported by Sarlin et al. (147). CAZymes were retrieved from https://mycocosm.jgi.doe.gov/mycocosm/home.

### Data availability.

The RNA-seq data generated in this work have been deposited in NCBI’s Gene Expression Omnibus (https://www.ncbi.nlm.nih.gov/geo) and BioProject (https://www.ncbi.nlm.nih.gov/bioproject) and are accessible through GEO series accession numbers GSE109088 (spore germination on medium data set), PRJNA849030 (spore germination on host data set), and PRJNA849358 (infected trichomes and epidermal cells data set).

## References

[1] Chandra Nayaka S, Hosahatti R, Prakash G, Tara Satyavathi C, Sharma R. 2021. Blast disease of cereal crops: evolution and adaptation in context of climate change. Springer Nature, London, UK.

[2] Asibi AE, Chai Q, Coulter JA. 2019. Rice blast: a disease with implications for global food security. Agronomy 9:451. doi:10.3390/agronomy9080451.

[3] Kazan K, Gardiner DM, Manners JM. 2012. On the trail of a cereal killer: recent advances in *Fusarium graminearum* pathogenomics and host resistance. Mol Plant Pathol 13:399–413. doi:10.1111/j.1364-3703.2011.00762.x. 22098555 PMC6638652

[4] Pasquali M, Beyer M, Logrieco A, Audenaert K, Balmas V, Basler R, Boutigny A-L, Chrpová J, Czembor E, Gagkaeva T, González-Jaén MT, Hofgaard IS, Köycü ND, Hoffmann L, Lević J, Marin P, Miedaner T, Migheli Q, Moretti A, Müller MEH, Munaut F, Parikka P, Pallez-Barthel M, Piec J, Scauflaire J, Scherm B, Stanković S, Thrane U, Uhlig S, Vanheule A, Yli-Mattila T, Vogelgsang S. 2016. A European database of *Fusarium graminearum* and *F. culmorum* trichothecene genotypes. Front Microbiol 7:406. doi:10.3389/fmicb.2016.00406. 27092107 PMC4821861

[5] Savary S, Willocquet L, Pethybridge SJ, Esker P, McRoberts N, Nelson A. 2019. The global burden of pathogens and pests on major food crops. Nat Ecol Evol 3:430–439. doi:10.1038/s41559-018-0793-y. 30718852

[6] Jansen C, von Wettstein D, Schäfer W, Kogel K-H, Felk A, Maier FJ. 2005. Infection patterns in barley and wheat spikes inoculated with wild-type and trichodiene synthase gene disrupted *Fusarium graminearum*. Proc Natl Acad Sci USA 102:16892–16897. doi:10.1073/pnas.0508467102. 16263921 PMC1283850

[7] Mentges M, Glasenapp A, Boenisch M, Malz S, Henrissat B, Frandsen RJN, Güldener U, Münsterkötter M, Bormann J, Lebrun M-H, Schäfer W, Martinez-Rocha AL. 2020. Infection cushions of *Fusarium graminearum* are fungal arsenals for wheat infection. Mol Plant Pathol 21:1070–1087. doi:10.1111/mpp.12960. 32573086 PMC7368127

[8] Dubin HJ. 1997. Fusarium head scab: global status and future prospects. *In* Proceedings of a Workshop Held at CIMMYT, El Batan, Mexico, 13–17 October, 1996. CIMMYT, El Batan, Mexico.

[9] Pestka JJ. 2007. Deoxynivalenol: toxicity, mechanisms and animal health risks. Animal Feed Science and Technology 137:283–298. doi:10.1016/j.anifeedsci.2007.06.006.

[10] Yang S, Li X, Chen W, Liu T, Zhong S, Ma L, Zhang M, Zhang H, Yu D, Luo P. 2016. Wheat resistance to *Fusarium* head blight is associated with changes in photosynthetic parameters. Plant Dis 100:847–852. doi:10.1094/PDIS-04-14-0398-RE. 30688616

[11] Bai G, Shaner G. 2004. Management and resistance in wheat and barley to *Fusarium* head blight. Annu Rev Phytopathol 42:135–161. doi:10.1146/annurev.phyto.42.040803.140340. 15283663

[12] Li W, Chern M, Yin J, Wang J, Chen X. 2019. Recent advances in broad-spectrum resistance to the rice blast disease. Curr Opin Plant Biol 50:114–120. doi:10.1016/j.pbi.2019.03.015. 31163394

[13] Velásquez AC, Castroverde CDM, He SY. 2018. Plant–pathogen warfare under changing climate conditions. Curr Biol 28:R619–R634. doi:10.1016/j.cub.2018.03.054. 29787730 PMC5967643

[14] Cuperlovic-Culf M, Vaughan M, Vermillion K, Surendra A, Teresi J, McCormick S. 2019. Effects of atmospheric CO level on the metabolic response of resistant and susceptible wheat to *Fusarium graminearum* infection. Mol Plant Microbe Interact 32:379–391. doi:10.1094/MPMI-06-18-0161-R. 30256178

[15] Manstretta V, Gourdain E, Rossi V. 2015. Deposition patterns of *Fusarium graminearum* ascospores and conidia within a wheat canopy. Eur J Plant Pathol 143:873–880. doi:10.1007/s10658-015-0722-8.

[16] Imboden L, Afton D, Trail F. 2018. Surface interactions of *Fusarium graminearum* on barley. Mol Plant Pathol 19:1332–1342. doi:10.1111/mpp.12616. 28940853 PMC6638126

[17] Beyer M, Röding S, Ludewig A, Verreet J-A. 2004. Germination and survival of *Fusarium graminearum* macroconidia as affected by environmental factors. J Phytopathology 152:92–97. doi:10.1111/j.1439-0434.2003.00807.x.

[18] Yi J, Hu Y, Zhang L, Yang G, Tang L. 2013. The effect of *Magnaporthe oryzae* conidia on photosystem in rice. IJESD 4:582–585. doi:10.7763/IJESD.2013.V4.417.

[19] Cruz CD, Kiyuna J, Bockus WW, Todd TC, Stack JP, Valent B. 2015. *Magnaporthe oryzae* conidia on basal wheat leaves as a potential source of wheat blast inoculum. Plant Pathol 64:1491–1498. doi:10.1111/ppa.12414.

[20] Sephton-Clark PCS, Voelz K. 2018. Spore germination of pathogenic filamentous fungi. Adv Appl Microbiol 102:117–157. doi:10.1016/bs.aambs.2017.10.002. 29680124

[21] Rogers LM, Flaishman MA, Kolattukudy PE. 1994. Cutinase gene disruption in *Fusarium solani* f. sp. *pisi* decreases its virulence on pea. Plant Cell 6:935–945. doi:10.1105/tpc.6.7.935. 8069105 PMC160490

[22] Woloshuk CP, Kolattukudy PE. 1986. Mechanism by which contact with plant cuticle triggers cutinase gene expression in the spores of *Fusarium solani* f. sp. *pisi*. Proc Natl Acad Sci USA 83:1704–1708. doi:10.1073/pnas.83.6.1704. 16593666 PMC323152

[23] Skamnioti P, Gurr SJ. 2008. Cutinase and hydrophobin interplay: a herald for pathogenesis? Plant Signal Behav 3:248–250. doi:10.4161/psb.3.4.5181. 19704644 PMC2634192

[24] Ryder LS, Talbot NJ. 2015. Regulation of appressorium development in pathogenic fungi. Curr Opin Plant Biol 26:8–13. doi:10.1016/j.pbi.2015.05.013. 26043436 PMC4781897

[25] Martin-Urdiroz M, Oses-Ruiz M, Ryder LS, Talbot NJ. 2016. Investigating the biology of plant infection by the rice blast fungus *Magnaporthe oryzae*. Fungal Genet Biol 90:61–68. doi:10.1016/j.fgb.2015.12.009. 26703899

[26] Kershaw MJ, Basiewicz M, Soanes DM, Yan X, Ryder LS, Csukai M, Oses-Ruiz M, Valent B, Talbot NJ. 2019. Conidial morphogenesis and septin-mediated plant infection require Smo1, a Ras GTPase-activating protein in *Magnaporthe oryzae*. Genetics 211:151–167. doi:10.1534/genetics.118.301490. 30446520 PMC6325701

[27] Franck WL, Gokce E, Randall SM, Oh Y, Eyre A, Muddiman DC, Dean RA. 2015. Phosphoproteome analysis links protein phosphorylation to cellular remodeling and metabolic adaptation during *Magnaporthe oryzae* appressorium development. J Proteome Res 14:2408–2424. doi:10.1021/pr501064q. 25926025 PMC4838196

[28] Franck WL, Gokce E, Oh Y, Muddiman DC, Dean RA. 2013. Temporal analysis of the *Magnaporthe oryzae* proteome during conidial germination and cyclic AMP (cAMP)-mediated appressorium formation. Mol Cell Proteomics 12:2249–2265. doi:10.1074/mcp.M112.025874. 23665591 PMC3734583

[29] Tucker SL, Talbot NJ. 2001. Surface attachment and pre-penetration stage development by plant pathogenic fungi. Annu Rev Phytopathol 39:385–417. doi:10.1146/annurev.phyto.39.1.385. 11701871

[30] Seong K-Y, Zhao X, Xu J-R, Güldener U, Kistler HC. 2008. Conidial germination in the filamentous fungus *Fusarium graminearum*. Fungal Genet Biol 45:389–399. doi:10.1016/j.fgb.2007.09.002. 17950638

[31] Aylor D. 2017. Chapter 3. Physical properties, forces, and processes affecting pollen and spores, in motion and at rest, p 31–45. *In* Aerial dispersal of pollen and spores. APS Publications, St. Paul, MN.

[32] Aylor D. 2017. Chapter 4. Release mechanisms (liberation), p 47–69. *In* Aerial Dispersal of Pollen and Spores. APS Publications, St. Paul, MN.

[33] Aylor D. 2017. Chapter 5. Deposition processes, p 71–94. *In* Aerial Dispersal of Pollen and Spores. APS Publications, St. Paul, MN.

[34] Peraldi A, Beccari G, Steed A, Nicholson P. 2011. *Brachypodium distachyon*: a new pathosystem to study *Fusarium* head blight and other *Fusarium* diseases of wheat. BMC Plant Biol 11:100. doi:10.1186/1471-2229-11-100. 21639892 PMC3123626

[35] Wilson RA, Talbot NJ. 2009. Under pressure: investigating the biology of plant infection by *Magnaporthe oryzae*. Nat Rev Microbiol 7:185–195. doi:10.1038/nrmicro2032. 19219052

[36] Eseola AB, Ryder LS, Osés-Ruiz M, Findlay K, Yan X, Cruz-Mireles N, Molinari C, Garduño-Rosales M, Talbot NJ. 2021. Investigating the cell and developmental biology of plant infection by the rice blast fungus *Magnaporthe oryzae*. Fungal Genet Biol 154:103562. doi:10.1016/j.fgb.2021.103562. 33882359

[37] Brown NA, Evans J, Mead A, Hammond-Kosack KE. 2017. A spatial temporal analysis of the *Fusarium graminearum* transcriptome during symptomless and symptomatic wheat infection. Molecular Plant Pathology 18:1295–1312. doi:10.1111/mpp.12564. 28466509 PMC5697668

[38] Biselli C, Bagnaresi P, Faccioli P, Hu X, Balcerzak M, Mattera MG, Yan Z, Ouellet T, Cattivelli L, Valè G. 2018. Comparative transcriptome profiles of near-isogenic hexaploid wheat lines differing for effective alleles at the 2DL FHB resistance QTL. Front Plant Sci 9:37. doi:10.3389/fpls.2018.00037. 29434615 PMC5797473

[39] Niu L, Pan L, Zeng W, Lu Z, Cui G, Fan M, Xu Q, Wang Z, Li G. 2018. Dynamic transcriptomes of resistant and susceptible peach lines after infestation by green peach aphids (*Myzus persicae* Sülzer) reveal defence responses controlled by the *Rm3* locus. BMC Genomics 19:846. doi:10.1186/s12864-018-5215-7. 30486776 PMC6264056

[40] Wang L, Li Q, Liu Z, Surendra A, Pan Y, Li Y, Zaharia LI, Ouellet T, Fobert PR. 2018. Integrated transcriptome and hormone profiling highlight the role of multiple phytohormone pathways in wheat resistance against fusarium head blight. PLoS One 13:e0207036. doi:10.1371/journal.pone.0207036. 30403737 PMC6221353

[41] Bönnighausen J, Schauer N, Schäfer W, Bormann J. 2019. Metabolic profiling of wheat rachis node infection by *Fusarium graminearum*—decoding deoxynivalenol-dependent susceptibility. New Phytol 221:459–469. doi:10.1111/nph.15377. 30084118

[42] Oh Y, Donofrio N, Pan H, Coughlan S, Brown DE, Meng S, Mitchell T, Dean RA. 2008. Transcriptome analysis reveals new insight into appressorium formation and function in the rice blast fungus *Magnaporthe oryzae*. Genome Biol 9:R85. doi:10.1186/gb-2008-9-5-r85. 18492280 PMC2441471

[43] Soanes DM, Chakrabarti A, Paszkiewicz KH, Dawe AL, Talbot NJ. 2012. Genome-wide transcriptional profiling of appressorium development by the rice blast fungus *Magnaporthe oryzae*. PLoS Pathog 8:e1002514. doi:10.1371/journal.ppat.1002514. 22346750 PMC3276559

[44] Mosquera G, Giraldo MC, Khang CH, Coughlan S, Valent B. 2009. Interaction transcriptome analysis identifies *Magnaporthe oryzae* BAS1-4 as biotrophy-associated secreted proteins in rice blast disease. Plant Cell 21:1273–1290. doi:10.1105/tpc.107.055228. 19357089 PMC2685627

[45] Shimizu M, Nakano Y, Hirabuchi A, Yoshino K, Kobayashi M, Yamamoto K, Terauchi R, Saitoh H. 2019. RNA-seq of *in planta*-expressed *Magnaporthe oryzae* genes identifies MoSVP as a highly expressed gene required for pathogenicity at the initial stage of infection. Mol Plant Pathol 20:1682–1695. doi:10.1111/mpp.12869. 31560822 PMC6859710

[46] Kim S, Park J, Park S-Y, Mitchell TK, Lee Y-H. 2010. Identification and analysis of *in planta* expressed genes of *Magnaporthe oryzae*. BMC Genomics 11:104. doi:10.1186/1471-2164-11-104. 20146797 PMC2832786

[47] Shi B-J, Wang G-L. 2008. Comparative study of genes expressed from rice fungus-resistant and susceptible lines during interactions with *Magnaporthe oryzae*. Gene 427:80–85. doi:10.1016/j.gene.2008.09.015. 18848973

[48] Wang Z, Miguel-Rojas C, Lopez-Giraldez F, Yarden O, Trail F, Townsend JP. 2019. Metabolism and development during conidial germination in response to a carbon-nitrogen-rich synthetic or a natural source of nutrition in *Neurospora crassa*. mBio 10:e00192-19. doi:10.1128/mBio.00192-19. 30914504 PMC6437048

[49] Wang Z, Gudibanda A, Ugwuowo U, Trail F, Townsend JP. 2018. Using evolutionary genomics, transcriptomics, and systems biology to reveal gene networks underlying fungal development. Fungal Biology Rev 32:249–264. doi:10.1016/j.fbr.2018.02.001.

[50] Wang Z, Lopez-Giraldez F, Lehr N, Farré M, Common R, Trail F, Townsend JP. 2014. Global gene expression and focused knockout analysis reveals genes associated with fungal fruiting body development in *Neurospora crassa*. Eukaryot Cell 13:154–169. doi:10.1128/EC.00248-13. 24243796 PMC3910948

[51] Trail F, Wang Z, Stefanko K, Cubba C, Townsend JP. 2017. The ancestral levels of transcription and the evolution of sexual phenotypes in filamentous fungi. PLoS Genet 13:e1006867. doi:10.1371/journal.pgen.1006867. 28704372 PMC5509106

[52] Sikhakolli UR, López-Giráldez F, Li N, Common R, Townsend JP, Trail F. 2012. Transcriptome analyses during fruiting body formation in *Fusarium graminearum* and *Fusarium verticillioides* reflect species life history and ecology. Fungal Genet Biol 49:663–673. doi:10.1016/j.fgb.2012.05.009. 22705880

[53] Kim W, Cavinder B, Proctor RH, O'Donnell K, Townsend JP, Trail F. 2019. Comparative genomics and transcriptomics during sexual development gives insight into the life history of the cosmopolitan fungus *Fusarium neocosmosporiellum*. Front Microbiol 10:1247. doi:10.3389/fmicb.2019.01247. 31231336 PMC6568001

[54] Kim W, Wang Z, Kim H, Pham K, Tu Y, Townsend JP, Trail F. 2022. Transcriptional divergence underpinning sexual development in the fungal class *Sordariomycetes*. mBio 13:e0110022. doi:10.1128/mbio.01100-22. 35638737 PMC9239162

[55] Lee YH, Dean RA. 1993. cAMP regulates infection structure formation in the plant pathogenic fungus *Magnaporthe grisea*. Plant Cell 5:693–700. doi:10.1105/tpc.5.6.693. 12271080 PMC160306

[56] Kim W, Miguel-Rojas C, Wang J, Townsend JP, Trail F. 2018. Developmental dynamics of long noncoding RNA expression during sexual fruiting body formation in *Fusarium graminearum*. mBio 9:e01292-18. doi:10.1128/mBio.01292-18. 30108170 PMC6094484

[57] Robinson MD, McCarthy DJ, Smyth GK. 2010. edgeR: a Bioconductor package for differential expression analysis of digital gene expression data. Bioinformatics 26:139–140. doi:10.1093/bioinformatics/btp616. 19910308 PMC2796818

[58] Veneault-Fourrey C, Barooah M, Egan M, Wakley G, Talbot NJ. 2006. Autophagic fungal cell death is necessary for infection by the rice blast fungus. Science 312:580–583. doi:10.1126/science.1124550. 16645096

[59] Sun G, Elowsky C, Li G, Wilson RA. 2019. TOR-autophagy branch signaling via Imp1 dictates plant-microbe biotrophic interface longevity. PLoS Genet 15:e1008016. doi:10.1371/journal.pgen.1008016. 30817760 PMC6394894

[60] Yun Y, Liu Z, Yin Y, Jiang J, Chen Y, Xu J, Ma Z. 2015. Functional analysis of the *Fusarium graminearum* phosphatome. New Phytol 207:119–134. doi:10.1111/nph.13374. 25758923

[61] Hou Z, Xue C, Peng Y, Katan T, Kistler HC, Xu J-R. 2002. A mitogen-activated protein kinase gene (MGV1) in *Fusarium graminearum* is required for female fertility, heterokaryon formation, and plant infection. Mol Plant Microbe Interact 15:1119–1127. doi:10.1094/MPMI.2002.15.11.1119. 12423017

[62] Segorbe D, Di Pietro A, Pérez-Nadales E, Turrà D. 2017. Three *Fusarium oxysporum* mitogen-activated protein kinases (MAPKs) have distinct and complementary roles in stress adaptation and cross-kingdom pathogenicity. Mol Plant Pathol 18:912–924. doi:10.1111/mpp.12446. 27301316 PMC6638227

[63] Bendtsen JD, Nielsen H, von Heijne G, Brunak S. 2004. Improved prediction of signal peptides: SignalP 3.0. J Molecular Biology 340:783–795. doi:10.1016/j.jmb.2004.05.028. 15223320

[64] Bendtsen JD, Jensen LJ, Blom N, Von Heijne G, Brunak S. 2004. Feature-based prediction of non-classical and leaderless protein secretion. Protein Eng Des Sel 17:349–356. doi:10.1093/protein/gzh037. 15115854

[65] Petre B, Kamoun S. 2014. How do filamentous pathogens deliver effector proteins into plant cells?. PLoS Biology 12:e1001801. 24586116 10.1371/journal.pbio.1001801 PMC3934835

[66] Thines E, Weber RW, Talbot NJ. 2000. MAP kinase and protein kinase A-dependent mobilization of triacylglycerol and glycogen during appressorium turgor generation by *Magnaporthe grisea*. Plant Cell 12:1703–1718. doi:10.1105/tpc.12.9.1703. 11006342 PMC149080

[67] Wang Z-Y, Thornton CR, Kershaw MJ, Debao L, Talbot NJ. 2003. The glyoxylate cycle is required for temporal regulation of virulence by the plant pathogenic fungus *Magnaporthe grisea*. Mol Microbiol 47:1601–1612. doi:10.1046/j.1365-2958.2003.03412.x. 12622815

[68] Wang Z-Y, Soanes DM, Kershaw MJ, Talbot NJ. 2007. Functional analysis of lipid metabolism in *Magnaporthe grisea* reveals a requirement for peroxisomal fatty acid beta-oxidation during appressorium-mediated plant infection. Mol Plant Microbe Interact 20:475–491. doi:10.1094/MPMI-20-5-0475. 17506326

[69] Bhambra GK, Wang Z-Y, Soanes DM, Wakley GE, Talbot NJ. 2006. Peroxisomal carnitine acetyl transferase is required for elaboration of penetration hyphae during plant infection by *Magnaporthe grisea*. Mol Microbiol 61:46–60. doi:10.1111/j.1365-2958.2006.05209.x. 16824094

[70] Ramos-Pamplona M, Naqvi NI. 2006. Host invasion during rice-blast disease requires carnitine-dependent transport of peroxisomal acetyl-CoA. Mol Microbiol 61:61–75. doi:10.1111/j.1365-2958.2006.05194.x. 16824095

[71] Ma Z, Song T, Zhu L, Ye W, Wang Y, Shao Y, Dong S, Zhang Z, Dou D, Zheng X, Tyler BM, Wang Y. 2015. A *Phytophthora sojae* glycoside hydrolase 12 protein is a major virulence factor during soybean infection and is recognized as a PAMP. Plant Cell 27:2057–2072. doi:10.1105/tpc.15.00390. 26163574 PMC4531360

[72] Gui Y-J, Chen J-Y, Zhang D-D, Li N-Y, Li T-G, Zhang W-Q, Wang X-Y, Short DPG, Li L, Guo W, Kong Z-Q, Bao Y-M, Subbarao KV, Dai X-F. 2017. *Verticillium dahliae* manipulates plant immunity by glycoside hydrolase 12 proteins in conjunction with carbohydrate-binding module 1. Environ Microbiol 19:1914–1932. doi:10.1111/1462-2920.13695. 28205292

[73] Quoc NB, Chau NNB. 2017. The role of cell wall degrading enzymes in pathogenesis of *Magnaporthe oryzae*. Curr Protein Pept Sci 18:1019–1034. doi:10.2174/1389203717666160813164955. 27526928

[74] Kikot GE, Hours RA, Alconada TM. 2009. Contribution of cell wall degrading enzymes to pathogenesis of *Fusarium graminearum*: a review. J Basic Microbiol 49:231–241. doi:10.1002/jobm.200800231. 19025875

[75] Rafiei V, Vélëz H, Tzelepis G. 2021. The role of glycoside hydrolases in phytopathogenic fungi and oomycetes virulence. Int J Mol Sci 22:9359. doi:10.3390/ijms22179359. 34502268 PMC8431085

[76] Brown HE, Esher SK, Alspaugh JA. 2020. Chitin: a “hidden figure” in the fungal cell wall. Curr Top Microbiol Immunol 425:83–111. doi:10.1007/82_2019_184. 31807896

[77] Gao F, Zhang B-S, Zhao J-H, Huang J-F, Jia P-S, Wang S, Zhang J, Zhou J-M, Guo H-S. 2019. Deacetylation of chitin oligomers increases virulence in soil-borne fungal pathogens. Nat Plants 5:1167–1176. doi:10.1038/s41477-019-0527-4. 31636399

[78] Chen S, Songkumarn P, Venu RC, Gowda M, Bellizzi M, Hu J, Liu W, Ebbole D, Meyers B, Mitchell T, Wang G-L. 2013. Identification and characterization of *in planta*-expressed secreted effector proteins from *Magnaporthe oryzae* that induce cell death in rice. Mol Plant Microbe Interact 26:191–202. doi:10.1094/MPMI-05-12-0117-R. 23035914

[79] Wösten HA. 2001. Hydrophobins: multipurpose proteins. Annu Rev Microbiol 55:625–646. doi:10.1146/annurev.micro.55.1.625. 11544369

[80] Lysøe E, Pasquali M, Breakspear A, Kistler HC. 2011. The transcription factor FgStuAp influences spore development, pathogenicity, and secondary metabolism in *Fusarium graminearum*. Mol Plant Microbe Interact 24:54–67. doi:10.1094/MPMI-03-10-0075. 20879840

[81] Quarantin A, Hadeler B, Kröger C, Schäfer W, Favaron F, Sella L, Martínez-Rocha AL. 2019. Different hydrophobins of *Fusarium graminearum* are involved in hyphal growth, attachment, water-air interface penetration and plant infection. Front Microbiol 10:751. doi:10.3389/fmicb.2019.00751. 31031728 PMC6474331

[82] Kim S, Ahn I-P, Rho H-S, Lee Y-H. 2005. MHP1, a *Magnaporthe grisea* hydrophobin gene, is required for fungal development and plant colonization. Mol Microbiol 57:1224–1237. doi:10.1111/j.1365-2958.2005.04750.x. 16101997

[83] Flynn CM, Broz K, Jonkers W, Schmidt-Dannert C, Kistler HC. 2019. Expression of the *Fusarium graminearum* terpenome and involvement of the endoplasmic reticulum-derived toxisome. Fungal Genet Biol 124:78–87. doi:10.1016/j.fgb.2019.01.006. 30664933 PMC6664814

[84] Boenisch MJ, Broz KL, Purvine SO, Chrisler WB, Nicora CD, Connolly LR, Freitag M, Baker SE, Kistler HC. 2017. Structural reorganization of the fungal endoplasmic reticulum upon induction of mycotoxin biosynthesis. Sci Rep 7:44296. doi:10.1038/srep44296. 28287158 PMC5347122

[85] Gardiner DM, Kazan K, Manners JM. 2009. Novel genes of *Fusarium graminearum* that negatively regulate deoxynivalenol production and virulence. Mol Plant Microbe Interact 22:1588–1600. doi:10.1094/MPMI-22-12-1588. 19888824

[86] Güldener U, Seong K-Y, Boddu J, Cho S, Trail F, Xu J-R, Adam G, Mewes H-W, Muehlbauer GJ, Kistler HC. 2006. Development of a *Fusarium graminearum* Affymetrix GeneChip for profiling fungal gene expression *in vitro* and *in planta*. Fungal Genet Biol 43:316–325. doi:10.1016/j.fgb.2006.01.005. 16531083

[87] Amarasinghe CC, Fernando WGD. 2016. Comparative analysis of deoxynivalenol biosynthesis related gene expression among different chemotypes of *Fusarium graminearum* in spring wheat. Front Microbiol 7:1229. doi:10.3389/fmicb.2016.01229. 27550207 PMC4976091

[88] Jiang C, Zhang C, Wu C, Sun P, Hou R, Liu H, Wang C, Xu J-R. 2016. TRI6 and TRI10 play different roles in the regulation of deoxynivalenol (DON) production by cAMP signalling in *Fusarium graminearum*. Environ Microbiol 18:3689–3701. doi:10.1111/1462-2920.13279. 26940955

[89] Seong K-Y, Pasquali M, Zhou X, Song J, Hilburn K, McCormick S, Dong Y, Xu J-R, Kistler HC. 2009. Global gene regulation by *Fusarium* transcription factors Tri6 and Tri10 reveals adaptations for toxin biosynthesis. Mol Microbiol 72:354–367. doi:10.1111/j.1365-2958.2009.06649.x. 19320833

[90] Anderson JM, Kim SW, Kopeček J. 2013. Advances in Drug Delivery Systems. *In* Anderson JM, Kim SW, Kopeček J (ed). 6: Proceedings of the Sixth International Symposium on Recent Advances in Drug Delivery Systems, Salt Lake City, UT, U.S.A., February 21-24, 1993. 1st Edition. Elsevier, Amsterdam, Netherlands.

[91] Kimura M, Shingu Y, Yoneyama K, Yamaguchi I. 1998. Features of*Tri101*, the trichothecene 3-*O*-acetyltransferase gene, related to the self-defense mechanism in *Fusarium graminearum*. Biosci Biotechnol Biochem 62:1033–1036. doi:10.1271/bbb.62.1033. 9648241

[92] McCormick SP, Alexander NJ. 2002. *Fusarium Tri8* encodes a trichothecene C-3 esterase. Appl Environ Microbiol 68:2959–2964. doi:10.1128/AEM.68.6.2959-2964.2002. 12039755 PMC123960

[93] Alexander NJ, McCormick SP, Hohn TM. 1999. TRI12, a trichothecene efflux pump from *Fusarium sporotrichioides*: gene isolation and expression in yeast. Mol Gen Genet 261:977–984. doi:10.1007/s004380051046. 10485289

[94] Lind AL, Lim FY, Soukup AA, Keller NP, Rokas A. 2018. An LaeA- and BrlA-dependent cellular network governs tissue-specific secondary metabolism in the human pathogen. mSphere 3:e00050-18. doi:10.1128/mSphere.00050-18. 29564395 PMC5853485

[95] Kumar D, Barad S, Chen Y, Luo X, Tannous J, Dubey A, Glam Matana N, Tian S, Li B, Keller N, Prusky D. 2017. LaeA regulation of secondary metabolism modulates virulence in *Penicillium expansum* and is mediated by sucrose. Mol Plant Pathol 18:1150–1163. doi:10.1111/mpp.12469. 27528575 PMC6638289

[96] Bok JW, Keller NP. 2016. 2. Insight into fungal secondary metabolism from ten years of LaeA research, p 21–29. *In* Hoffmeister D (ed). Biochemistry and molecular biology. The mycota, vol 3. Springer, Cham, Switzerland.

[97] Saha P, Ghosh S, Roy-Barman S. 2020. *MoLAEA* regulates secondary metabolism in *Magnaporthe oryzae*. mSphere 5:e00936-19. doi:10.1128/mSphere.00936-19. 32238572 PMC7113587

[98] Kim H-K, Lee S, Jo S-M, McCormick SP, Butchko RAE, Proctor RH, Yun S-H. 2013. Functional roles of FgLaeA in controlling secondary metabolism, sexual development, and virulence in *Fusarium graminearum*. PLoS One 8:e68441. doi:10.1371/journal.pone.0068441. 23874628 PMC3713025

[99] Jia L-J, Tang H-Y, Wang W-Q, Yuan T-L, Wei W-Q, Pang B, Gong X-M, Wang S-F, Li Y-J, Zhang D, Liu W, Tang W-H. 2019. A linear nonribosomal octapeptide from *Fusarium graminearum* facilitates cell-to-cell invasion of wheat. Nat Commun 10:922. doi:10.1038/s41467-019-08726-9. 30804501 PMC6389888

[100] Boedi S, Berger H, Sieber C, Münsterkötter M, Maloku I, Warth B, Sulyok M, Lemmens M, Schuhmacher R, Güldener U, Strauss J. 2016. Comparison of *Fusarium graminearum* transcriptomes on living or dead wheat differentiates substrate-responsive and defense-responsive genes. Front Microbiol 7:1113. doi:10.3389/fmicb.2016.01113. 27507961 PMC4960244

[101] Keller N, Palmer J, Bayram O. 2017. Fungal jewels: secondary metabolites. Frontiers Media doi:10.3389/978-2-88945-136-4.

[102] Skellam E. 2021. Analysis of the secondary metabolism in *Magnaporthe oryzae*. Methods Mol Biol 2356:41–56. doi:10.1007/978-1-0716-1613-0_3. 34236675

[103] He M, Xu Y, Chen J, Luo Y, Lv Y, Su J, Kershaw MJ, Li W, Wang J, Yin J, Zhu X, Liu X, Chern M, Ma B, Wang J, Qin P, Chen W, Wang Y, Wang W, Ren Z, Wu X, Li P, Li S, Peng Y, Lin F, Talbot NJ, Chen X. 2018. MoSnt2-dependent deacetylation of histone H3 mediates MoTor-dependent autophagy and plant infection by the rice blast fungus *Magnaporthe oryzae*. Autophagy 14:1543–1561. doi:10.1080/15548627.2018.1458171. 29929416 PMC6135590

[104] Kershaw MJ, Talbot NJ. 2009. Genome-wide functional analysis reveals that infection-associated fungal autophagy is necessary for rice blast disease. Proc Natl Acad Sci USA 106:15967–15972. doi:10.1073/pnas.0901477106. 19717456 PMC2747227

[105] Talbot NJ, Kershaw MJ. 2009. The emerging role of autophagy in plant pathogen attack and host defence. Curr Opin Plant Biol 12:444–450. doi:10.1016/j.pbi.2009.05.008. 19625208

[106] Lv W, Wang C, Yang N, Que Y, Talbot NJ, Wang Z. 2017. Genome-wide functional analysis reveals that autophagy is necessary for growth, sporulation, deoxynivalenol production and virulence in *Fusarium graminearum*. Sci Rep 7:11062. doi:10.1038/s41598-017-11640-z. 28894236 PMC5594004

[107] Nguyen LN, Bormann J, Le GTT, Stärkel C, Olsson S, Nosanchuk JD, Giese H, Schäfer W. 2011. Autophagy-related lipase FgATG15 of *Fusarium graminearum* is important for lipid turnover and plant infection. Fungal Genet Biol 48:217–224. doi:10.1016/j.fgb.2010.11.004. 21094265

[108] Guenther JC, Trail F. 2005. The development and differentiation of *Gibberella zeae* (anamorph: *Fusarium graminearum*) during colonization of wheat. Mycologia 97:229–237. doi:10.3852/mycologia.97.1.229. 16389974

[109] Baggaley LE. 2021. How fungal pathogens communicate with plant cells and cause disease. PhD dissertation. University of Exeter, Exeter, UK.

[110] Boenisch MJ, Schäfer W. 2011. *Fusarium graminearum* forms mycotoxin producing infection structures on wheat. BMC Plant Biol 11:110. doi:10.1186/1471-2229-11-110. 21798058 PMC3166921

[111] Valent B. 2021. The impact of blast disease: past, present, and future. Methods Mol Biol 2356:1–18. doi:10.1007/978-1-0716-1613-0_1. 34236673

[112] Sakulkoo W, Osés-Ruiz M, Oliveira Garcia E, Soanes DM, Littlejohn GR, Hacker C, Correia A, Valent B, Talbot NJ. 2018. A single fungal MAP kinase controls plant cell-to-cell invasion by the rice blast fungus. Science 359:1399–1403. doi:10.1126/science.aaq0892. 29567712

[113] Sun G, Qi X, Wilson RA. 2019. A feed-forward subnetwork emerging from integrated TOR- and cAMP/PKA-signaling architecture reinforces appressorium morphogenesis. Mol Plant Microbe Interact 32:593–607. doi:10.1094/MPMI-10-18-0287-R. 30431400

[114] Marroquin-Guzman M, Sun G, Wilson RA. 2017. Glucose-ABL1-TOR signaling modulates cell cycle tuning to control terminal appressorial cell differentiation. PLoS Genet 13:e1006557. doi:10.1371/journal.pgen.1006557. 28072818 PMC5266329

[115] Marroquin-Guzman M, Wilson RA. 2015. GATA-dependent glutaminolysis drives appressorium formation in *Magnaporthe oryzae* by suppressing TOR inhibition of cAMP/PKA signaling. PLoS Pathog 11:e1004851. doi:10.1371/journal.ppat.1004851. 25901357 PMC4406744

[116] Yu F, Gu Q, Yun Y, Yin Y, Xu J, Shim W, Ma Z. 2014. The TOR signaling pathway regulates vegetative development and virulence in *Fusarium graminearum*. New Phytol 203:219–232. doi:10.1111/nph.12776. 24684168

[117] Liu Z, Liu N, Jiang H, Yan L, Ma Z, Yin Y. 2018. The activators of type 2A phosphatases (PP2A) regulate multiple cellular processes via PP2A-dependent and -independent mechanisms in *Fusarium graminearum*. Mol Plant Microbe Interact 31:1121–1133. doi:10.1094/MPMI-03-18-0056-R. 29877164

[118] Mogg C, Bonner C, Wang L, Schernthaner J, Smith M, Desveaux D, Subramaniam R. 2019. Genomic identification of the TOR signaling pathway as a target of the plant alkaloid antofine in the phytopathogen *Fusarium graminearum*. mBio 10:e00792-19. doi:10.1128/mBio.00792-19. 31186319 PMC6561021

[119] Talas F, Kalih R, Miedaner T, McDonald BA. 2016. Genome-wide association study identifies novel candidate genes for aggressiveness, deoxynivalenol production, and azole sensitivity in natural field populations of *Fusarium graminearum*. Mol Plant Microbe Interact 29:417–430. doi:10.1094/MPMI-09-15-0218-R. 26959837

[120] Dean RA, Talbot NJ, Ebbole DJ, Farman ML, Mitchell TK, Orbach MJ, Thon M, Kulkarni R, Xu J-R, Pan H, Read ND, Lee Y-H, Carbone I, Brown D, Oh YY, Donofrio N, Jeong JS, Soanes DM, Djonovic S, Kolomiets E, Rehmeyer C, Li W, Harding M, Kim S, Lebrun M-H, Bohnert H, Coughlan S, Butler J, Calvo S, Ma L-J, Nicol R, Purcell S, Nusbaum C, Galagan JE, Birren BW. 2005. The genome sequence of the rice blast fungus *Magnaporthe grisea*. Nature 434:980–986. doi:10.1038/nature03449. 15846337

[121] Cuomo CA, Güldener U, Xu J-R, Trail F, Turgeon BG, Di Pietro A, Walton JD, Ma L-J, Baker SE, Rep M, Adam G, Antoniw J, Baldwin T, Calvo S, Chang Y-L, Decaprio D, Gale LR, Gnerre S, Goswami RS, Hammond-Kosack K, Harris LJ, Hilburn K, Kennell JC, Kroken S, Magnuson JK, Mannhaupt G, Mauceli E, Mewes H-W, Mitterbauer R, Muehlbauer G, Münsterkötter M, Nelson D, O'Donnell K, Ouellet T, Qi W, Quesneville H, Roncero MIG, Seong K-Y, Tetko IV, Urban M, Waalwijk C, Ward TJ, Yao J, Birren BW, Kistler HC. 2007. The *Fusarium graminearum* genome reveals a link between localized polymorphism and pathogen specialization. Science 317:1400–1402. doi:10.1126/science.1143708. 17823352

[122] King R, Urban M, Hammond-Kosack MCU, Hassani-Pak K, Hammond-Kosack KE. 2015. The completed genome sequence of the pathogenic ascomycete fungus *Fusarium graminearum*. BMC Genomics 16:544. doi:10.1186/s12864-015-1756-1. 26198851 PMC4511438

[123] Kazan K, Gardiner DM. 2018. Transcriptomics of cereal-*Fusarium graminearum* interactions: what we have learned so far. Mol Plant Pathol 19:764–778. doi:10.1111/mpp.12561. 28411402 PMC6638174

[124] Skadsen RW, Hohn TM. 2004. Use of *Fusarium graminearum* transformed with gfp to follow infection patterns in barley and *Arabidopsis*. Physiol Mol Plant Path 64:45–53. doi:10.1016/j.pmpp.2004.04.003.

[125] Kim KW. 2019. Plant trichomes as microbial habitats and infection sites. Eur J Plant Pathol 154:157–169. doi:10.1007/s10658-018-01656-0.

[126] Hamer JE, Howard RJ, Chumley FG, Valent B. 1988. A mechanism for surface attachment in spores of a plant pathogenic fungus. Science 239:288–290. doi:10.1126/science.239.4837.288. 17769992

[127] Godfray HCJ, Mason-D’Croz D, Robinson S. 2016. Food system consequences of a fungal disease epidemic in a major crop. Philos Trans R Soc Lond B Biol Sci 371:20150467. doi:10.1098/rstb.2015.0467. 28080990 PMC5095543

[128] Trail F, Common R. 2000. Perithecial development by *Gibberella zeae*: a light microscopy study. Mycologia 92:130–138. doi:10.2307/3761457.

[129] Metzenberg RL. 2004. Bird medium: an alternative to Vogel medium. Fungal Genet Rep 51:19–20. doi:10.4148/1941-4765.1138.

[130] Cappellini RA, Peterson JL. 1965. Macroconidium formation in submerged cultures by a non-sporulating strain of *Gibberella zeae*. Mycologia 57:962–966. doi:10.2307/3756895.

[131] Talbot NJ, Ebbole DJ, Hamer JE. 1993. Identification and characterization of *MPG1*, a gene involved in pathogenicity from the rice blast fungus *Magnaporthe grisea*. Plant Cell 5:1575–1590. doi:10.1105/tpc.5.11.1575. 8312740 PMC160387

[132] Lewandowski SM, Bushnell WR, Evans CK. 2006. Distribution of mycelial colonies and lesions in field-grown barley inoculated with *Fusarium graminearum*. Phytopathology 96:567–581. doi:10.1094/PHYTO-96-0567. 18943174

[133] Zadoks JC, Chang TT, Konzak CF. 1974. A decimal code for the growth stages of cereals. Weed Res 14:415–421. doi:10.1111/j.1365-3180.1974.tb01084.x.

[134] Hong YS, Kang S, Han M, Gobert GN, Jones MK. 2011. High quality RNA isolation from *Aedes aegypti* midguts using laser microdissection microscopy. Parasit Vectors 4:83. doi:10.1186/1756-3305-4-83. 21595925 PMC3121693

[135] Gupta VK, Pandey BK. 2013. Histopathological technique for detection of fungal infections in plants, p 197–200. *In* Gupta V, Tuohy M, Ayyachamy M, Turner K, O’Donovan A (ed). Laboratory protocols in fungal biology. Springer, New York, NY.

[136] Shabman BC. 1943. Tannic acid and iron alum with Safranin and Orange G in studies of the shoot apex. Stain Technol 18:105–111. doi:10.3109/10520294309105799.

[137] Lillie RD, Conn HJ, Biological Stain Commission. 1991. HJ Conn’s biological stains. Sigma Chemical Co., St. Louis, MO.

[138] Chen C, Khaleel SS, Huang H, Wu CH. 2014. Software for pre-processing Illumina next-generation sequencing short read sequences. Source Code Biol Med 9:8. doi:10.1186/1751-0473-9-8. 24955109 PMC4064128

[139] Kim D, Langmead B, Salzberg SL. 2015. HISAT: a fast spliced aligner with low memory requirements. Nat Methods 12:357–360. doi:10.1038/nmeth.3317. 25751142 PMC4655817

[140] Li H, Handsaker B, Wysoker A, Fennell T, Ruan J, Homer N, Marth G, Abecasis G, Durbin R, 1000 Genome Project Data Processing Subgroup. 2009. The sequence alignment/map format and SAMtools. Bioinformatics 25:2078–2079. doi:10.1093/bioinformatics/btp352. 19505943 PMC2723002

[141] Pertea M, Pertea GM, Antonescu CM, Chang T-C, Mendell JT, Salzberg SL. 2015. StringTie enables improved reconstruction of a transcriptome from RNA-seq reads. Nat Biotechnol 33:290–295. doi:10.1038/nbt.3122. 25690850 PMC4643835

[142] Anders S, Pyl PT, Huber W. 2015. HTSeq—a Python framework to work with high-throughput sequencing data. Bioinformatics 31:166–169. doi:10.1093/bioinformatics/btu638. 25260700 PMC4287950

[143] Gotz S, Garcia-Gomez JM, Terol J, Williams TD, Nagaraj SH, Nueda MJ, Robles M, Talon M, Dopazo J, Conesa A. 2008. High-throughput functional annotation and data mining with the Blast2GO suite. Nucleic Acids Res 36:3420–3435. doi:10.1093/nar/gkn176. 18445632 PMC2425479

[144] Priebe S, Kreisel C, Horn F, Guthke R, Linde J. 2015. FungiFun2: a comprehensive online resource for systematic analysis of gene lists from fungal species. Bioinformatics 31:445–446. doi:10.1093/bioinformatics/btu627. 25294921 PMC4308660

[145] Tralamazza SM, Rocha LO, Oggenfuss U, Corrêa B, Croll D. 2019. Complex evolutionary origins of specialized metabolite gene cluster diversity among the plant pathogenic fungi of the *Fusarium graminearum* species complex. Genome Biol Evol 11:3106–3122. doi:10.1093/gbe/evz225. 31609418 PMC6836718

[146] Villafana R, Ramdass A, Rampersad S. 2019. Selection of *Fusarium* trichothecene toxin genes for molecular detection depends on *TRI* gene cluster organization and gene function. Toxins 11:36. doi:10.3390/toxins11010036. 30646506 PMC6357111

[147] Sarlin T, Kivioja T, Kalkkinen N, Linder MB, Nakari-Setälä T. 2012. Identification and characterization of gushing-active hydrophobins from *Fusarium graminearum* and related species. J Basic Microbiol 52:184–194. doi:10.1002/jobm.201100053. 21780148

