# Supplementary figures and images for: Comparative Transcriptomics of Fusarium graminearum and Magnaporthe oryzae Spore Germination Leading up To Infection
Source: mBio. 2023 Jan 4;14(1):e02442-22. doi: 10.1128/mbio.02442-22 (PMC9973345; doi:10.1128/mbio.02442-22)

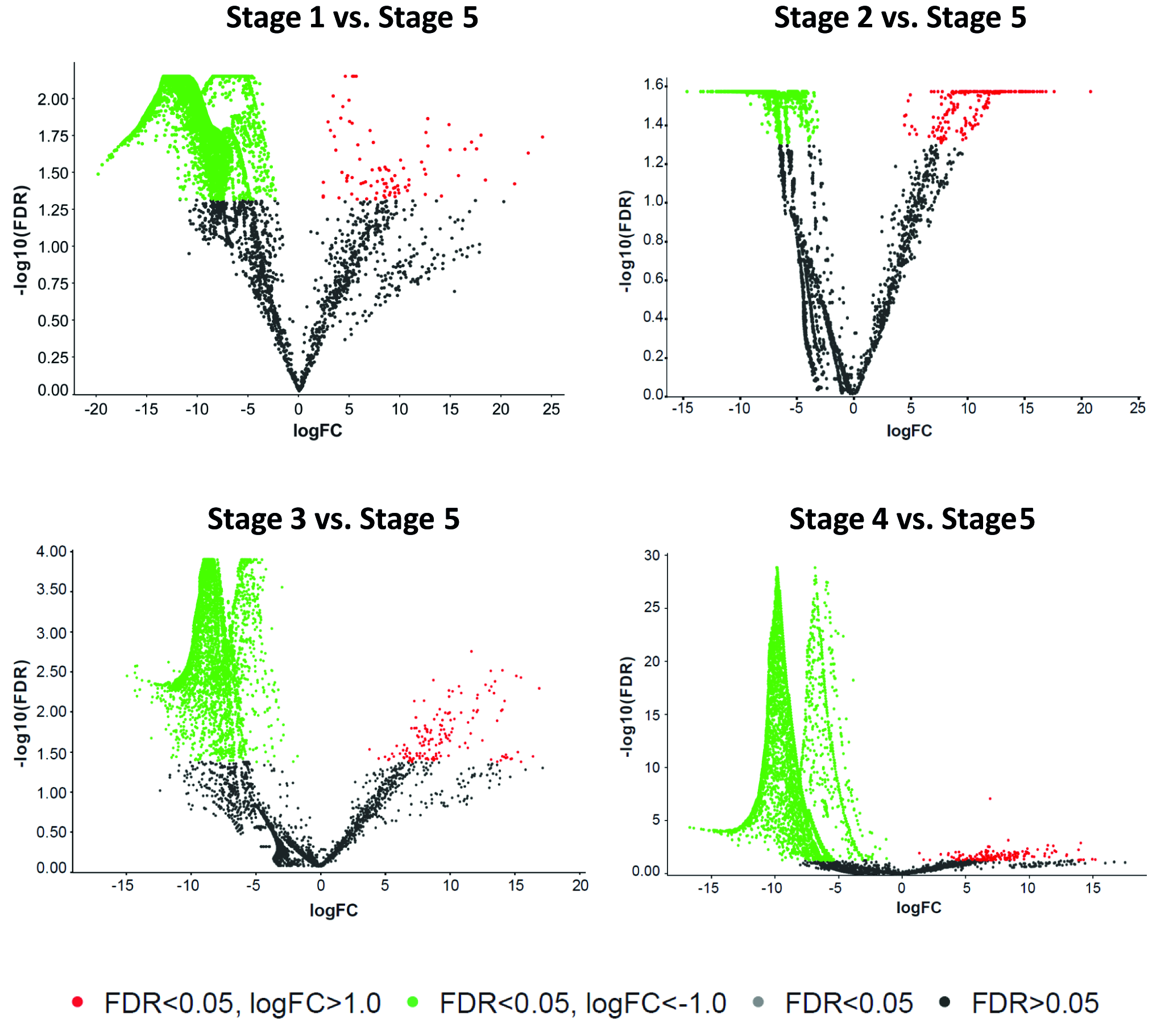

Supplement: FIG S1 [file mbio.02442-22-s0001.tif]

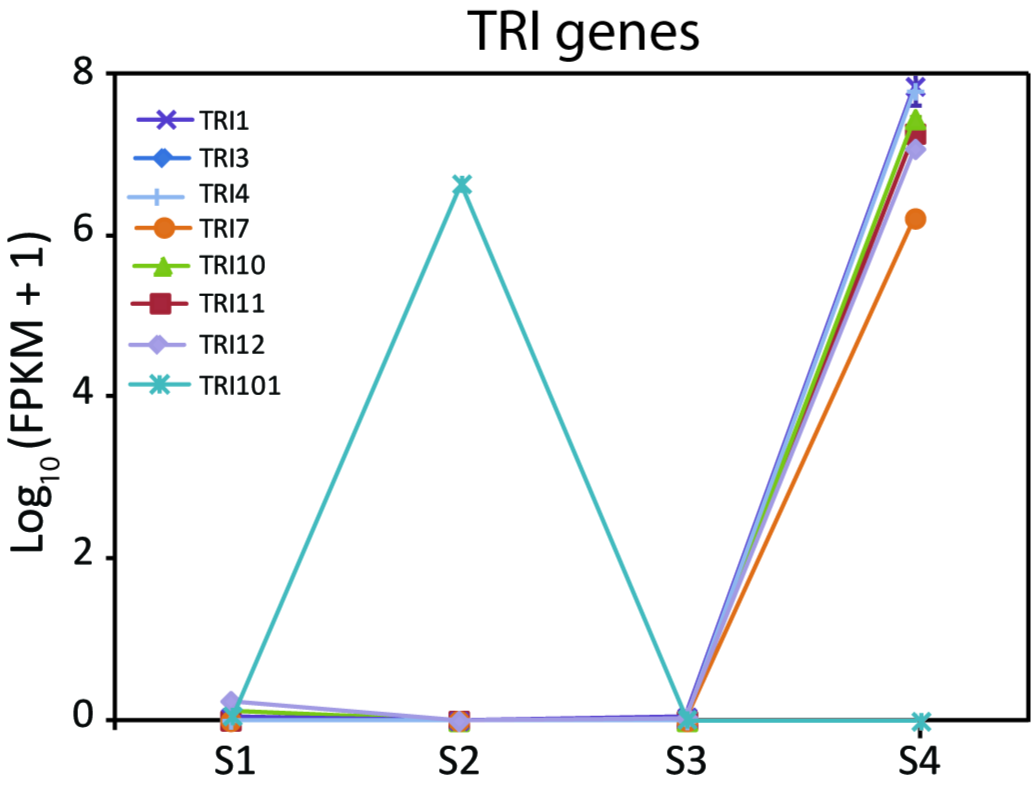

Supplement: FIG S2 [file mbio.02442-22-s0002.tif]

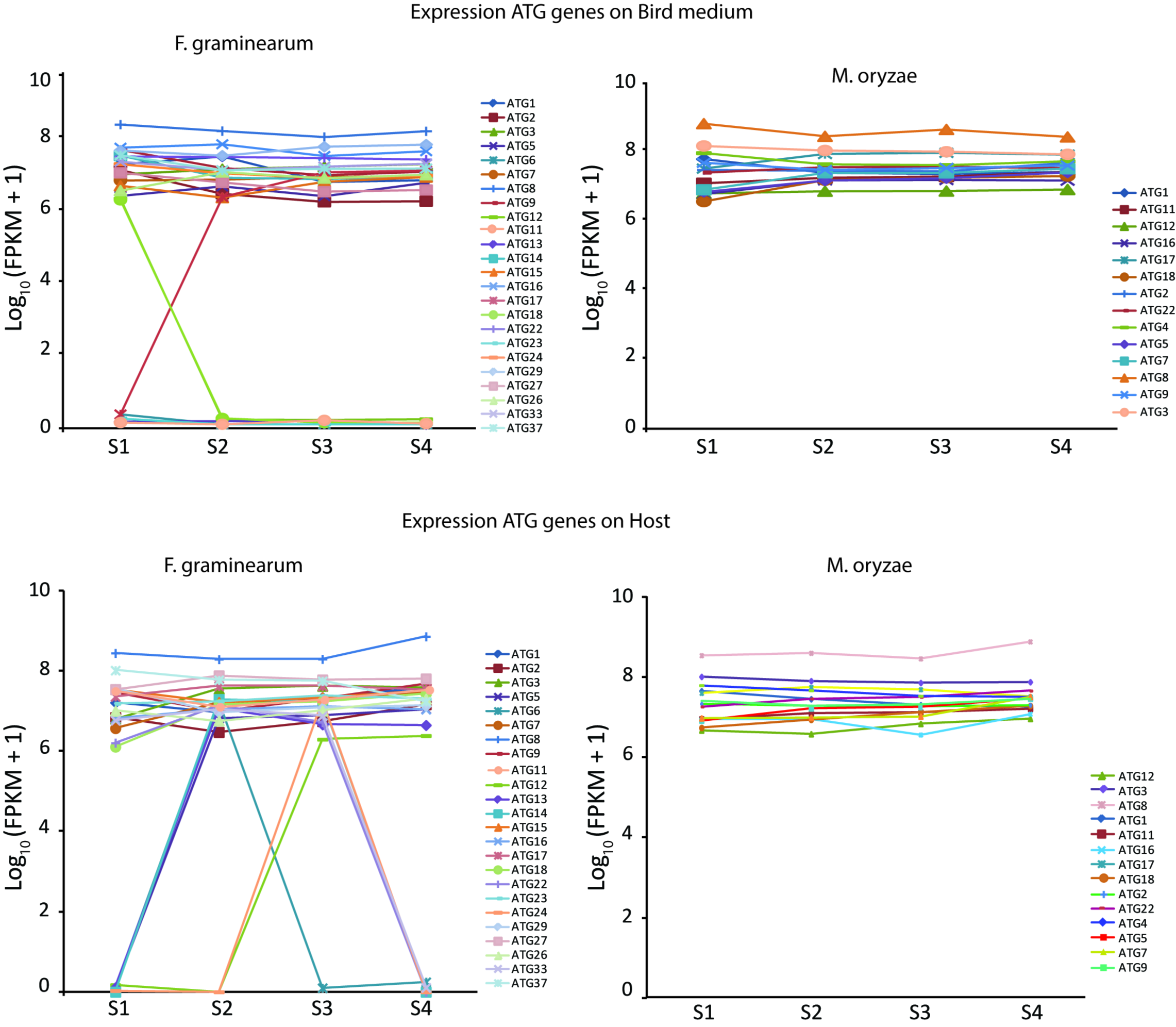

Supplement: FIG S3 [file mbio.02442-22-s0003.tif]
